# Nutrient and Rainfall Additions Shift Phylogenetically Estimated Traits of Soil Microbial Communities

**DOI:** 10.3389/fmicb.2017.01271

**Published:** 2017-07-11

**Authors:** Kelly Gravuer, Anu Eskelinen

**Affiliations:** ^1^Graduate Group in Ecology, Department of Plant Sciences, University of California, Davis Davis, CA, United States; ^2^Department of Physiological Diversity, Helmholtz Center for Environmental Research–UFZ Leipzig, Germany; ^3^German Centre for Integrative Biodiversity Research (iDiv) Halle-Jena-Leipzig Leipzig, Germany; ^4^Department of Ecology, University of Oulu Oulu, Finland; ^5^Department of Environmental Science and Policy, University of California, Davis Davis, CA, United States

**Keywords:** traits, rRNA gene copy number, genome size, California, grassland, serpentine, fertilization, climate change

## Abstract

Microbial traits related to ecological responses and functions could provide a common currency facilitating synthesis and prediction; however, such traits are difficult to measure directly for all taxa in environmental samples. Past efforts to estimate trait values based on phylogenetic relationships have not always distinguished between traits with high and low phylogenetic conservatism, limiting reliability, especially in poorly known environments, such as soil. Using updated reference trees and phylogenetic relationships, we estimated two phylogenetically conserved traits hypothesized to be ecologically important from DNA sequences of the 16S rRNA gene from soil bacterial and archaeal communities. We sampled these communities from an environmental change experiment in California grassland applying factorial addition of late-season precipitation and soil nutrients to multiple soil types for 3 years prior to sampling. Estimated traits were rRNA gene copy number, which contributes to how rapidly a microbe can respond to an increase in resources and may be related to its maximum growth rate, and genome size, which suggests the breadth of environmental and substrate conditions in which a microbe can thrive. Nutrient addition increased community-weighted mean estimated rRNA gene copy number and marginally increased estimated genome size, whereas precipitation addition decreased these community means for both estimated traits. The effects of both treatments on both traits were associated with soil properties, such as ammonium, available phosphorus, and pH. Estimated trait responses within several phyla were opposite to the community mean response, indicating that microbial responses, although largely consistent among soil types, were not uniform across the tree of life. Our results show that phylogenetic estimation of microbial traits can provide insight into how microbial ecological strategies interact with environmental changes. The method could easily be applied to any of the thousands of existing 16S rRNA sequence data sets and offers potential to improve our understanding of how microbial communities mediate ecosystem function responses to global changes.

## Introduction

A central goal of microbial ecology is to understand how environmental factors shape microbial community composition and function, and to use that knowledge to predict how microbial communities will respond to environmental change. The past decade has seen advances toward this goal as high-throughput DNA sequencing has enabled more comprehensive microbial censuses. Much of this data is taxonomic marker gene sequences (Lane et al., 1985; Gilbert et al., 2014), which furnish little information about ecological behavior. Some progress has been made by delineating broad microbial clades with roughly similar ecology, but many microbes do not fit easily into such groupings (Fierer et al., 2007; Philippot et al., 2010; Koeppel and Wu, 2012).

To facilitate comparison, synthesis, and prediction with microbial composition data sets, a common currency is needed. Such a currency could also aid in incorporating microbes into biogeochemical models, an advance that holds promise for improving model predictions (Bouskill et al., 2012; Treseder et al., 2012; Powell et al., 2015; Treseder and Lennon, 2015; Wieder et al., 2015; Pagel et al., 2016), but that may prove difficult to achieve using taxonomic composition data alone (Graham et al., 2016).

Community-wide data on ecologically important traits—as summarized by metrics, such as community-weighted means—has provided such a currency for plant communities (McGill et al., 2006; Westoby and Wright, 2006) and could potentially do so for microbial communities as well (Goberna et al., 2014; Krause et al., 2014; Martiny et al., 2015; Treseder and Lennon, 2015). But in complex environments, such as soils, only a small percentage of taxa have been cultured, making direct measurement of community-wide traits very challenging. One alternative is to measure a microbe's response to an environmental change as a trait in itself. This approach has yielded unique insights (Evans and Wallenstein, 2014; Martiny et al., 2015; Amend et al., 2016), but because these responses are system-specific, their utility for predicting responses of communities with different initial compositions is somewhat limited. A second alternative is to infer community-wide trait values from metagenomic data, which has also produced exciting advances (Vieira-Silva and Rocha, 2010; Fierer et al., 2014; Leff et al., 2015; Nayfach and Pollard, 2015). However, metagenomic data are more expensive to obtain and more challenging to analyze than taxonomic markers and thus comprise a smaller proportion of existing microbial data sets, limiting the power of cross-data set syntheses. To complement these approaches, a reliable method for estimating microbial traits from taxonomic marker data is needed.

One strategy currently in use estimates the full content of each taxon's genome, using the placement of its marker sequence on a phylogenetic tree of fully sequenced reference genomes (Langille et al., 2013; Aßhauer et al., 2015; Bowman and Ducklow, 2015). However, because traits vary in their manner and speed of evolution, traits of neighboring taxa will produce better predictions for some traits than for others (Martiny et al., 2012, 2015; Goberna and Verdú, 2016). This limitation can be addressed by estimating only traits that evolve relatively slowly—that is, traits for which values of neighboring taxa are likely to provide reliable estimates (Goberna and Verdú, 2016).

Two microbial traits that are both relatively slowly evolving and hypothesized to be ecologically important are rRNA gene copy number and genome size. A higher number of rRNA gene copies suggests an ability to more rapidly increase growth in response to an increase in resources and may also support a higher maximum growth rate, but more copies may be disadvantageous under consistently low resource conditions (Klappenbach et al., 2000; Stevenson and Schmidt, 2004; Green et al., 2008; Lauro et al., 2009; Vieira-Silva and Rocha, 2010; Krause et al., 2014; Roller et al., 2016). Accordingly, microbes with higher rRNA gene copy numbers tend to be more abundant in environments where resources are found at higher levels and/or have more pulsed dynamics (Shrestha et al., 2007; Goldfarb et al., 2011; Goberna et al., 2014; Vuono et al., 2014; DeAngelis et al., 2015; Männistö et al., 2016; Nemergut et al., 2016). Importantly, microbes with many rRNA gene copies may also differ from those with few copies in the rate and efficiency with which they decompose organic matter, with consequences for soil carbon storage (Wieder et al., 2015). However, as there may be many genetic pathways to rapid growth or resource-use efficiency and our knowledge of the ecology of uncultured microbes is still limited (Buerger et al., 2012), additional evaluation is needed to determine whether and how different patterns of resource availability favor microbes with higher or lower numbers of rRNA gene copies.

Microbes with large genomes are expected to thrive in environments that are variable (e.g., with periods of aerobic and anaerobic conditions) and/or have high resource complexity or diversity, while microbes with small genomes tend to dominate in relatively constant environments with relatively few types of easy-to-metabolize resources (Vieira-Silva and Rocha, 2010; Guieysse and Wuertz, 2012; Barberán et al., 2014; Fierer et al., 2014; Giovannoni et al., 2014; Krause et al., 2014). In the context of environmental change, microbes with large genomes may be more resilient in the face of changing conditions (Guieysse and Wuertz, 2012; Barberán et al., 2014). Interestingly, some studies report reduced genome size in environments low in N and/or P, possibly to enhance replication efficiency (Giovannoni et al., 2014), while experimental addition of the same resources has been found to favor microbes with smaller genomes (Leff et al., 2015). Clearly more research is needed to elucidate the relationships between environmental changes and microbial traits.

Building on a published phylogenetic trait estimation method (Kembel et al., 2012), we estimated rRNA gene copy number and genome size values for soil bacterial and archaeal (hereafter, “microbial”) communities in an environmental change experiment. In this experiment, factorial additions of late-season precipitation and soil nutrients were applied to three adjacent grassland soil types for 3 years prior to our sampling. This design allowed us to test whether addition of two different resources (water and nutrients) would have similar effects on estimated microbial traits, as well as how these effects might interact and vary across soil types. Our samples captured both the direct effects of resource addition on the microbial community and indirect effects mediated by changes in plant biomass and composition, providing understanding of longer-term outcomes. Using community-weighted means to summarize estimated trait values, we addressed the following questions:

1. How will soil type, nutrient addition, late-season precipitation addition, and their interactions affect (a) community-weighted mean estimated rRNA gene copy number and (b) community-weighted mean estimated genome size?
2. Exploring potential variation in responses among groups within the community, how will community-weighted mean estimated trait changes within individual phyla compare to community-wide responses?

## Methods

### Field experiment design

The experiment was conducted at the University of California McLaughlin Reserve in the Northern Coast Ranges of California (N 38°52′, W122°26′), which has a Mediterranean climate. Rainfall averages 62 cm per year, falling mainly between November and March. Rainfall was below average in the year samples were collected and the preceding year (53.9 cm in 2012; 51.9 cm in 2013), with associated reductions in plant productivity (Copeland et al., 2016).

The ~1,000 × 500 m experimental site contains three distinct soil and grassland types (Eskelinen and Harrison, 2015c). The first two are underlain by ultramafic (serpentinite, peridotite) bedrock, which has a low Ca:Mg ratio and high levels of some heavy metals; these are generally referred to as “serpentine soils.” One type of serpentine soil (“harsh serpentine”) is shallow, coarse-textured, and low in organic matter and nutrients (Eskelinen and Harrison, 2014). This soil supports low-productivity plant communities of diverse native annual forbs and geophytes (Eskelinen and Harrison, 2015c). The second type of serpentine soil (“lush serpentine”) is deeper, finer-textured, and has higher levels of organic matter and nutrients—although its Ca:Mg ratio is comparable to harsh serpentine (Eskelinen and Harrison, 2014). It supports a higher-productivity plant community of native forbs and geophytes, scattered native perennial grasses, and exotic annual grasses and forbs (Eskelinen and Harrison, 2015c). The third type of soil (“non-serpentine”) is derived from sedimentary bedrock and is also deeper than the harsh serpentine, with a loamy texture and higher levels of organic matter and nutrients (Eskelinen and Harrison, 2014). Plant productivity in this soil is similar to that on the lush serpentine, and plant communities consist primarily of exotic annuals (grasses and forbs) (Eskelinen and Harrison, 2015c). Hereafter, these three different soil + associated grassland types will be referred to simply as “soil types.”

Environmental manipulations were imposed beginning in 2010. A “precipitation addition” treatment was designed to simulate a lengthening of the rainy season into the early summer, a regional scenario predicted by some climate change forecasts (National Assessment Synthesis Team, 2000). A “nutrient addition” treatment was designed to investigate the impact of this precipitation change under relaxed nutrient limitation. The full factorial treatment combination (precipitation added, nutrients added, both added, neither added) was applied to 10–12 replicate 2 × 2 m plots on each of the three soil types, for a total of 132 plots. In each year, precipitation addition began after the natural spring rains had ceased and was added to simulate one moderate storm event per week for 8 weeks. For nutrient addition, a slow-release granular NPK (10-10-10) fertilizer with micronutrients (Lilly Miller Ultra Green; Lilly Miller Brands, Walnut Creek, CA, USA) was broadcast in three equal applications in November, early February, and late March of each year, for a total of 10 g N/m^2^, 10 g P/m^2^, and 10 g K/m^2^ per year. See Eskelinen and Harrison (2014, 2015a,b,c) for further details of treatment implementation.

### Soil sample collection and microbial processing

Soil samples were collected for microbial community characterization on 28 and 29 May 2013. Sampling occurred 6 days after the last simulated rainfall of the year, capturing the season-long effect of the treatment. After brushing aside leaf litter to expose the mineral soil surface, soil cores (7 cm diameter) were taken to a depth of 7.5 cm. This was the deepest core that could be reliably collected in the harsh serpentine plots. In each plot, three cores were collected from a 1 × 1 m quadrat. Cores were composited and a subsample for DNA extraction was removed into a sterile tube, placed on ice, transported to the laboratory, and stored at −20°C. Remaining soil was placed on ice packs and stored at 4°C for <48 h before extraction with 0.5 M K_2_SO_4_ for measurement of ammonium (NH_4_-N), nitrate (NO_3_-N), and dissolved organic carbon (DOC). 10 g soil and 50 mL K_2_SO_4_ were shaken for 1 h at 175 rpm before filtering the extract through pre-leached Whatman #1 filter paper; from these extracts, ${\text{NH}}_{4}^{+}$ and ${\text{NO}}_{3}^{-}$ were measured colorimetrically (Kempers and Kok, 1989; Doane and Horwath, 2003) and dissolved organic carbon was measured with a Shimadzu Total Organic Carbon Analyzer (TOC-V CSH). Soil moisture was measured by comparing mass of a subsample before and after drying to constant mass at 105°C. Remaining soil was air-dried and sent to the A&L Western Laboratory (Modesto, CA) for measurement of additional soil chemical parameters, including pH, organic matter, Olsen P, S, K, Mg, Ca, Na, and cation exchange capacity (Gavlak et al., 2005). For testing associations between soil properties and estimated trait values, we also calculated two ratios: “extractable N:P” [(NH_4_-N + NO_3_-N)/Olsen P] and Ca:Mg.

DNA was extracted using a MoBio PowerLyzer kit (MoBio Laboratories, Carlsbad, CA). Extracted DNA was sent to the Argonne National Laboratory (Lemont, IL) for amplification and sequencing of the V4 region of the 16S rRNA gene. Amplification was performed following Earth Microbiome Project protocols (www.earthmicrobiome.org/emp-standard-protocols/16s), with 515F/806R primers from Caporaso et al. (2012). Paired-end 250 bp sequencing of the amplicon was performed on a MiSeq, in a run including only these 132 samples. Sequences are deposited in the Sequence Read Archive under accession number SRP098483.

### Clustering sequences into operational taxonomic units (OTUs)

Raw forward and reverse reads were demultiplexed (but not quality filtered) using QIIME 1.9.1 (Caporaso et al., 2010b). OTUs were then derived using the UPARSE pipeline in USEARCH 8.1 (Edgar, 2013). After forward and reverse reads were merged with USEARCH, all reads that had more than 1 barcode difference during the demultiplexing process were removed. To generate the set of high-quality sequences that would be used for picking OTUs, remaining merged reads were then quality filtered using a maximum error threshold (maxee) of 0.5 and a minimum length of 100. These filtered sequences were dereplicated and then clustered into OTUs at 97% similarity, removing singletons and chimeras in the process. Finally, any OTU that could not be mapped to greengenes (version 13_8) (DeSantis et al., 2006) with an identity of 75% or greater was discarded (Leff et al., 2015), which eliminated 6.8% of OTUs. The product of these processes was a set of 18,023 OTUs.

The full set of merged reads was then mapped to this OTU set to make the raw OTU table. Taxonomy was assigned to OTUs using the RDP classifier (Wang et al., 2007) implemented in QIIME 1.9.1. The raw OTU table was then filtered to remove all OTUs identified as chloroplasts or mitochondria. Next, the OTU table was rarefied to 26,690 sequences per sample (retaining 129 of the 132 plots). 100 rarefactions at this depth were performed in QIIME 1.9.1, and the 100 resulting tables were averaged.

### Constructing new reference tree, assessing accuracy, and estimating trait values

All statistical analyses were conducted in R (R Core Team, 2016). For bacterial and archaeal genomes, 16S rRNA gene sequences and annotated trait values were downloaded from the Joint Genome Institute's Integrated Microbial Genomes (IMG) database (Markowitz et al., 2012) on December 18, 2015. 16S rRNA sequences were aligned using PyNAST (Caporaso et al., 2010a) in QIIME 1.9.1, and trees were constructed with RAxML 8.2.4 (Stamatakis, 2014) using a GAMMA model of rate heterogeneity. Four candidate traits were initially selected for testing: rRNA gene copy number, genome size, oxygen requirement, and motility. Phylogenetic signal was assessed for each trait (via *K* and λ for continuous traits and *D* for binary traits) (Pagel, 1999; Blomberg et al., 2003; Fritz and Purvis, 2010) using the phytools and caper packages (Revell, 2012; Orme et al., 2013). To assess trait estimation accuracy, we used leave-one-out cross-validation (Kembel et al., 2012), as well as a more conservative “test species” method emphasizing taxa likely to be found in soil. We note that this trait estimation approach is inherently limited by the phylogenetic distribution of available fully-sequenced genomes (Table S1) vs. the phylogenetic distribution of soil microbes (e.g., Tables 1, 2). Bearing this caveat in mind, we believe that useful insight into the ecology of soil microbial communities can still be gained from trait estimation based on published genomes (e.g., Goberna et al., 2014; DeAngelis et al., 2015; Nemergut et al., 2016), and we encourage future studies on this topic to update our reference trees with newly-published genomes, just as we endeavored to update trees from previous studies here. See Supplementary Material for more details on tree construction and trait estimation testing.

**Table 1:** Changes in phylum relative abundances with soil types and treatments, with Proteobacteria shown by Class and Actinobacteria by Order.

**“Phylum”**	**% on Harsh**	**Soil preference**	**% change +nutrients**	**Statistical change +nutrients**	**% change +precip**	**Statistical change +precip**
Euryarchaeota	68.2	Harsh	−71.8	**decrease**	−30.2	
FBP	59.7	Harsh	−25.9		−13.7	
[Parvarchaeota]	59.0	Harsh	−47.0	**decrease**	41.2	**increase**
Actinobacteria: 0319-7L14	56.9	Harsh	−38.8	**decrease**	18.2	
OD1	52.3	Harsh	−18.3		68.6	**increase**
OP3	47.4	Harsh	−27.8		28.5	
Nitrospirae	45.3	Harsh	48.0	**increase**	20.2	**increase**
Actinobacteria: Acidimicrobiales	41.7	Harsh	−24.8	**decrease**	0.8	
Acidobacteria	40.0	Harsh	−5.2		−3.3	
Chlamydiae	39.9	Harsh	22.8		76.2	**increase**
Actinobacteria: Rubrobacterales	40.9	Harsh, Lush	−27.5	**decrease**	15.5	
Chlorobi	38.0	Harsh, Lush	10.4		24.5	**increase**
Bacteroidetes	27.6	Lush	22.3	**increase**	−7.8	
Actinobacteria: Actinomycetales	24.5	Lush	35.0	**increase**	−19.7	**decrease**
Fibrobacteres	22.3	Lush	174.2	**increase**	22.9	
Actinobacteria: Micrococcales	21.3	Lush	−11.3		67.2	**increase**
Betaproteobacteria	25.8	Lush, Non	36.2	**increase**	−21.4	**decrease**
Deltaproteobacteria	25.8	Lush, Non	18.6	**increase**	28.7	**increase**
Gammaproteobacteria	19.2	Lush, Non	60.1	**increase**	47.1	**increase**
WS3	14.9	Lush, Non	15.1		47.5	**increase**
Verrucomicrobia	29.2	Non	−11.0	**decrease**	−9.4	**decrease**
Alphaproteobacteria	28.7	Non	14.5	**increase**	−11.9	**decrease**
Firmicutes	22.6	Non	5.7		−38.8	**decrease**
Crenarchaeota	44.3	–	−9.7		61.1	**increase**
Cyanobacteria	43.0	–	−12.1		94.6	**increase**
Actinobacteria: Gaiellales	41.9	–	−19.5	**decrease**	26.2	**increase**
Actinobacteria: Solirubrobacterales	36.7	–	−18.1	**decrease**	−1.0	
TM7	35.5	–	−7.0		9.9	
Gemmatimonadetes	35.3	–	7.6		17.5	**increase**
BRC1	34.0	–	−5.3		5.0	
Armatimonadetes	33.8	–	−8.5		−21.8	**decrease**
Elusimicrobia	33.3	–	7.8		0.9	
Chloroflexi	32.7	–	−10.2	**decrease**	7.4	
Planctomycetes	32.0	–	−7.5		2.9	
TM6	30.5	–	43.9		171.9	**increase**
Tenericutes	26.7	–	−29.1	**decrease**	−36.9	**decrease**

*Phyla are grouped by soil preference, then by descending % on harsh serpentine. “Harsh” refers to harsh serpentine, “Lush” to lush serpentine, and “Non” to non-serpentine. Statistical changes indicate p < 0.05 main effects from models analyzing effects of soil type, nutrient treatment, precipitation treatment, and all interactions on relative abundance of each phylum, controlling for false discovery rate per Benjamini and Yekutieli (2001). Significant decreases in relative abundance are highlighted in red and significant increases in relative abundance are highlighted in green. Phyla with ≤ 0.01% relative abundance are not shown*.

**Table 2:** Changes in estimated trait values within phyla

**Phylum % relative abundance**	**“Phylum”**	**rRNA copy number**		**Genome size**	
		**Nutrient addition**	**Precipitation addition**	**Nutrient addition**	**Precipitation addition**
16.92	Acidobacteria				
11.51	Verrucomicrobia	**decrease**		**decrease**	**decrease**
10.99	Alphaproteobacteria			**decrease**	
9.09	Actinobacteria: Solirubrobacterales			**increase**	**increase**
7.60	Bacteroidetes	**decrease**	**decrease**		**increase**
5.62	Betaproteobacteria	**decrease**	**increase**	**increase**	**decrease**
5.05	Gemmatimonadetes				
4.72	Planctomycetes				
4.36	Actinobacteria: Rubrobacterales				**decrease**
3.72	Actinobacteria: Actinomycetales				
3.59	Chloroflexi				**increase**
2.99	Actinobacteria: Gaiellales				
2.97	Deltaproteobacteria	**increase**		**increase**	**decrease**
2.71	Crenarchaeota				
2.31	Gammaproteobacteria			**increase**	**decrease**
1.68	Actinobacteria: Acidimicrobiales			**decrease**	
0.63	Armatimonadetes		**increase**		**increase**
0.49	Elusimicrobia				
0.48	Chlorobi				
0.29	Actinobacteria: 0319-7L14				
0.29	Nitrospirae	**decrease**		**increase**	
0.21	Cyanobacteria	**decrease**	**decrease**	**decrease**	**decrease**
0.18	FBP	**decrease**		**decrease**	
0.18	Tenericutes				**decrease**
0.18	TM7				**decrease**
0.12	TM6				
0.11	Euryarchaeota				
0.10	Chlamydiae				
0.09	BRC1		**decrease**		
0.09	Fibrobacteres		**increase**		**decrease**
0.09	Actinobacteria: Micrococcales				
0.08	OD1				
0.08	WS3				
0.07	OP3				
0.05	Firmicutes				**increase**
0.04	[Parvarchaeota]				

*Proteobacteria are shown by Class and Actinobacteria by Order to highlight ecological differences within these abundant phyla. Phyla are shown in descending order of relative abundance across all plots and those with ≤ 0.01% are not shown. Significance values used to determine treatment effects represent main effects from models analyzing effects of soil type, nutrient treatment, precipitation treatment, and all interactions on the community-weighted mean estimated trait values for each phylum in each plot. Statistically significant (p < 0.05) decreases in trait values are highlighted in red and statistically significant increases in trait values are highlighted in green. The method of Benjamini and Yekutieli (2001) was used to control false discovery rates*.

Trait values were estimated for all experimental OTUs by placing them onto the reference tree using pplacer (Matsen et al., 2010), then using ancestral state estimation methods (Kembel et al., 2012) to calculate trait value estimates for each experimental OTU based on its phylogenetic position in relation to reference taxa (via picante:phyEstimate for continuous traits and picante:phyEstimateDisc for binary traits; Kembel et al., 2010). Using the rRNA gene copy number estimates, relative abundances in the OTU table were adjusted with the script from Kembel et al. (2012). Community weighted mean trait values were then calculated for each trait in each plot with the FD package (Laliberté and Legendre, 2010; Laliberté et al., 2014) using this adjusted OTU table. As a final test of the estimation procedure, we evaluated differences between community weighted mean estimated trait values calculated using the full set of OTUs vs. those calculated excluding OTUs with the least certain 20% of estimates. Considering all tests, estimation for two candidate traits was most robust: rRNA gene copy number and genome size. Only those traits were used in the analysis and inference below. Reference trees and associated trait values for reference taxa are available for download as Supplementary Material.

### Statistical modeling

#### Question 1: effects of soil type and treatments on community-weighted mean trait values

To test whether the experimental treatments affected community-weighted mean trait values, we used nlme (Pinheiro et al., 2016) to build linear mixed effects (lme) models with precipitation treatment, nutrient treatment, soil type, and all of their interactions as fixed effects and irrigation line as a random effect to reflect random error by line. Contrasts were implemented using the multcomp package (Hothorn et al., 2008). Because the estimated traits were only moderately correlated with one another in this study (*r* = 0.31) [and in a previous study using direct measurement in isolates (*r* = 0.35) (Klappenbach et al., 2000)], the analysis focused on each trait independently rather than a multivariate trait measure (but see Roller et al., 2016).

To explore the potential role of soil properties in mediating estimated microbial trait responses to precipitation and nutrient treatments, we re-ran the lme models with each of the measured soil chemical parameters, in turn, as a covariate. Covariates that caused a substantial decrease in a treatment's statistical significance would be candidates for mediating that treatment's effects on the estimated trait, although further experiments to isolate changes in these soil properties would be necessary to establish their role. In addition, we calculated Pearson correlations between individual soil properties and community-weighted mean estimated trait values across all plots.

To complement the treatment effects analysis, we also asked whether trait values of OTUs that increased in relative abundance in response to a treatment differed significantly from trait values of OTUs that decreased. For each treatment, we identified the OTUs that were present in both the control and treatment plots (pooled across soils), with “present” defined as having mean relative abundance >1 (out of 26,690), thus filtering out very rare taxa whose ecological responses may not have been adequately captured by our sequencing (Evans and Wallenstein, 2014). For each OTU in this set, we calculated the percentage change in mean relative abundance between the control and treatment plots. We then identified “increaser” OTUs as those in the highest quartile of relative abundance changes (corresponding to OTUs that increased by ~25% or more) and “decreaser” OTUs as those in the lowest quartile (corresponding to OTUs that decreased by ~25% or more), and we compared estimated trait values of the increasers and decreasers using a two-tailed *t*-test. Our results were robust to different thresholds for defining “increaser” and “decreaser” (see Results). We recognize that, because our data represents *relative* abundances, OTUs appearing to “decrease” might be maintaining their abundance or even increasing in absolute terms while other OTUs in the community show greater degrees of increase (and vice versa for apparent increasers). Nevertheless, we believe that these relative response differences represent meaningful distinctions.

#### Question 2: variation in trait responses among phyla

Taxonomic assignments from the RDP Classifier were used to group OTUs into phyla. Two large phyla that had the highest experiment-wide relative abundances (Proteobacteria and Actinobacteria) were split into lower taxonomic groups for this analysis (classes for Proteobacteria and orders for Actinobacteria), as these lower taxonomic groups have been shown to have distinct ecological behavior (Goodfellow and Williams, 1983; Fierer et al., 2007; Philippot et al., 2010; Cruz-Martínez et al., 2012). To determine whether phylum relative abundances changed in response to soil types and treatments, the relative abundance of each phylum in each plot was used as a response variable in the linear mixed model described above. To determine whether trait values *within* phyla responded to soil types and treatments, community-weighted mean estimated trait values were calculated for each phylum in each plot and used as response variables in the linear mixed model described above. Within both sets of analyses (phylum relative abundances and phylum-specific community-weighted mean trait values), the method of Benjamini and Yekutieli (2001) was used to control false discovery rates.

## Results

### Performance of trait estimation method

Both rRNA gene copy number and genome size had significant phylogenetic signal as measured by the *K* and λ metrics (*p* ≤ 0.001 in all cases, detailed in Supplementary Material 1.3). Across our two testing procedures, correlations between estimated and actual trait values were 0.87–0.90 for rRNA gene copy number and 0.90–0.92 for genome size. Comparing linear mixed effects model results for community-weighted means calculated using the full set of OTUs vs. for those calculated using only the 80% of OTUs with most certain trait estimates, the two methods had very similar results for both traits, suggesting that model fits were not driven by the least certain trait estimates (Table S2). See Supplementary Material for more detail on trait estimation performance, including actual vs. estimated trait values for 100 test species (Table S3).

### Effects of soil type on community-weighted trait means

**Figure 1:**
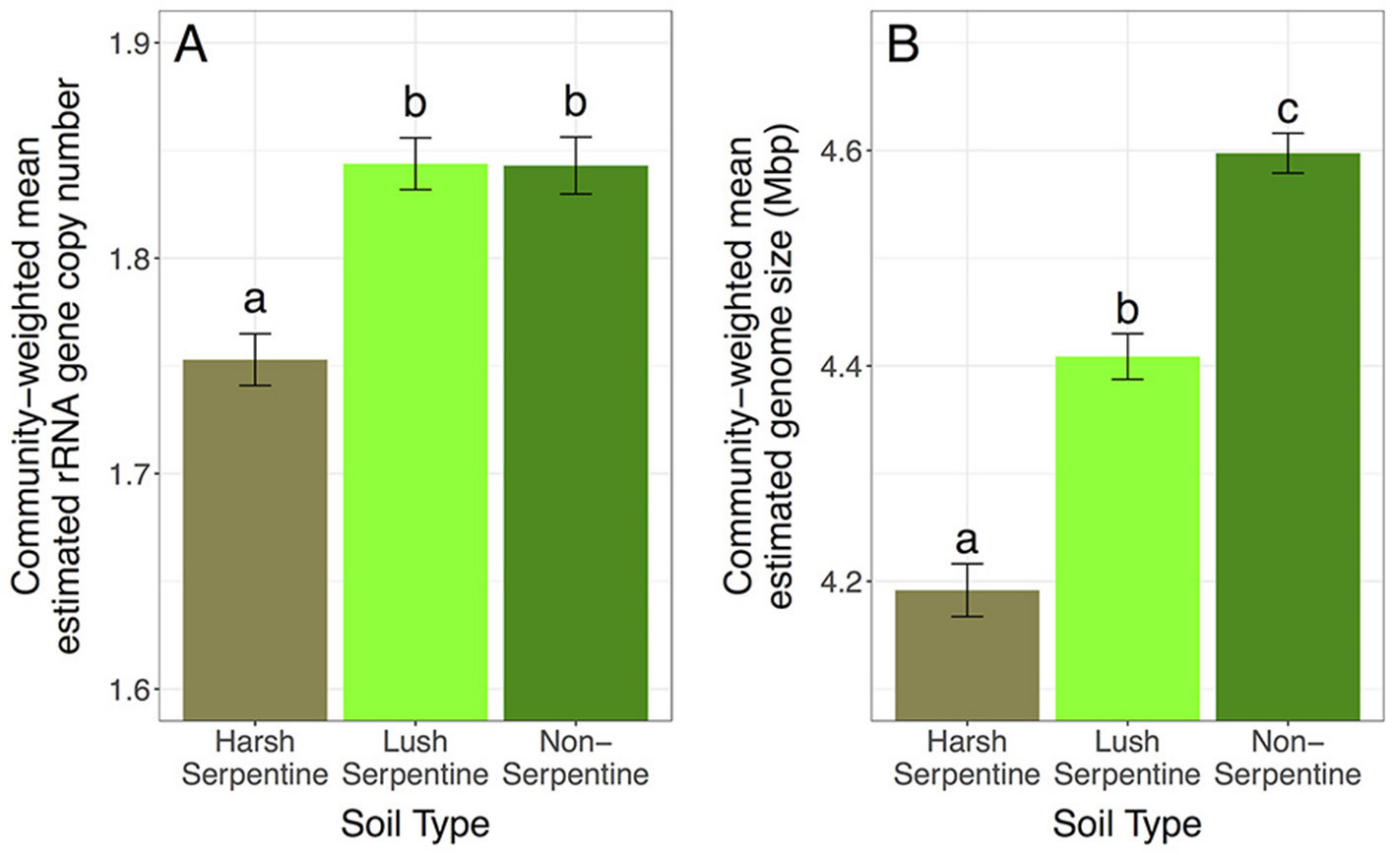
Main effect of soil type on community-weighted mean estimated traits, pooled across treatments: **(A)** estimated rRNA gene copy number, **(B)** estimated genome size. Error bars show 1 SE below mean and 1 SE above mean. Bars sharing a letter are not significantly different (at α = 0.05). The *y*-axes are scaled to highlight variation among soils, as it is not possible to have 0 rRNA gene copies or a 0 Mbp genome.

Community-weighted means for both estimated rRNA gene copy and estimated genome size differed among soils. Specifically, harsh serpentine microbial communities had a higher proportion of taxa with few estimated rRNA gene copies and small estimated genome sizes, with 4.93 and 4.89% lower community-weighted mean estimated rRNA gene copy numbers and 4.92 and 8.83% lower community-weighted mean estimated genome sizes than communities from the lush serpentine and non-serpentine soils, respectively [rRNA gene copy number: *F*_(2, 109)_ = 17.3, *p* < 0.0001; genome size: *F*_(2, 109)_ = 70.4, *p* < 0.0001] (Figure 1). Communities from the lush serpentine and non-serpentine soils had similar community-weighted mean estimated rRNA gene copy numbers (*p* = 0.984), while the non-serpentine had a 4.28% greater community-weighted mean estimated genome size than the lush serpentine (*p* < 0.0001).

### Effects of late-season precipitation and nutrient addition on community-weighted estimated trait means and trait value differences between “increaser” and “decreaser” taxa

**Figure 2:**
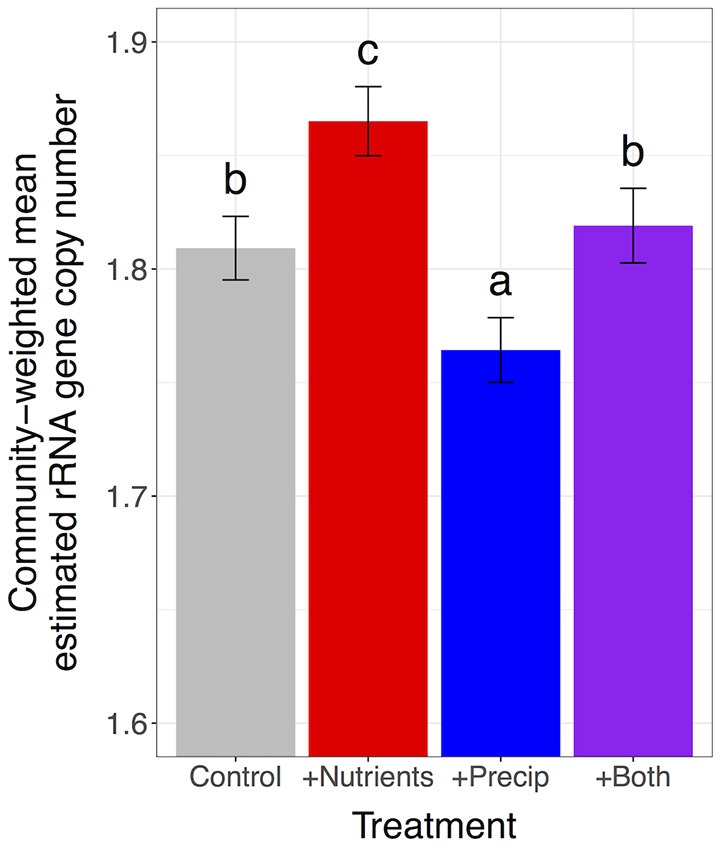
Main effects of nutrient addition and precipitation addition treatments on community-weighted mean estimated rRNA gene copy number. Error bars show 1 SE below mean and 1 SE above mean. The *y*-axis is scaled to highlight variation among treatments, as it is not possible to have 0 rRNA gene copies. Plots were pooled across soils since neither soil type x treatment interaction (nor the three-way interaction) was significant.

**Figure 3:**
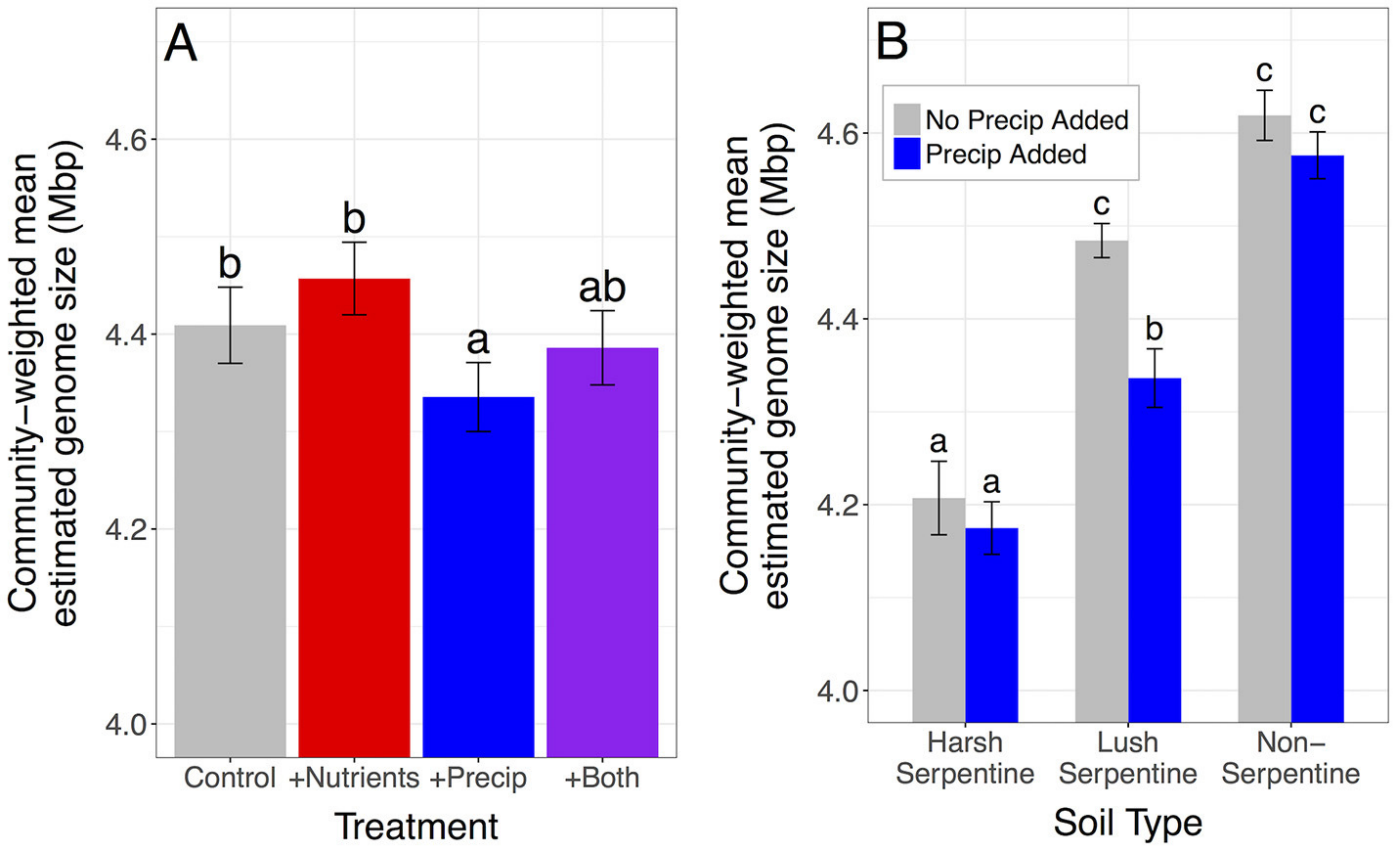
Main effects of nutrient addition and precipitation addition treatments on community-weighted mean estimated genome size. Neither the soil type x nutrient addition nor the three-way interaction was significant, but the soil type x precipitation addition interaction was borderline (*p* = 0.084). Therefore, genome size results are presented both ways: once with plots pooled across soils **(A)** and once showing the interaction **(B)**. Error bars show 1 SE below mean and 1 SE above mean. The *y*-axis is scaled to highlight variation among treatments, as it is not possible to have a 0 Mbp genome. Panel **(B)** suggests that main effect of precipitation addition on community-weighted mean estimated genome size was driven primarily by the lush serpentine soil.

Nutrient addition increased the proportion of microbes with high estimated rRNA gene copy numbers and marginally increased the proportion of microbes with large estimated genome sizes, increasing community-weighted means by 3.04% for estimated rRNA gene copy number and by 1.09% for estimated genome size, compared to unfertilized plots [rRNA gene copy number: *F*_(1, 109)_ = 18.3, *p* < 0.0001; genome size: *F*_(1, 109)_ = 3.3, *p* = 0.071] (Figures 2, 3). In contrast, late-season precipitation addition favored microbes with lower estimated copy numbers and smaller estimated genome sizes, decreasing community-weighted means by 2.40% for estimated rRNA gene copy number and by 1.61% for estimated genome size, compared to unwatered plots [rRNA gene copy number: *F*_(1, 109)_ = 13.5, *p* = 0.0004; genome size: *F*_(1, 109)_ = 10.9, *p* = 0.001] (Figures 2, 3). Both effects were largely consistent across soil types, although precipitation addition led to a marginally stronger reduction in community-weighted mean estimated genome size in the lush serpentine (3.30%) compared to the other two soil types (0.93% in non-serpentine and 0.78% in harsh serpentine) [soil type × precipitation treatment interaction: *F*_(2, 109)_ = 2.53, *p* = 0.084; watered vs. unwatered: lush serpentine *p* = 0.0004, harsh serpentine *p* = 0.821, non-serpentine *p* = 0.661] (Figure 3B). There was no interaction between the two treatments for either trait, nor was there three-way interaction among the two treatments and soil type (all *p* > 0.10).

OTUs that increased in relative abundance in response to nutrient addition had higher estimated rRNA gene copy numbers [1.99 ± 0.03 vs. 1.86 ± 0.03, *t*_(2155)_ = −3.26, *p* = 0.001] and larger estimated genome sizes [4.47 ± 0.07 vs. 4.19 ± 0.06, *t*_(2142)_ = −3.06, *p* = 0.002] than OTUs that decreased, on average (Figures S1, S2). Conversely, OTUs that increased in relative abundance in response to precipitation addition had lower estimated rRNA gene copy numbers [1.87 ± 0.02 vs. 1.96 ± 0.03, *t*_(2194)_ = 2.23, *p* = 0.026] and smaller estimated genome sizes [4.07 ± 0.06 vs. 4.42 ± 0.06, *t*_(2243)_ = 3.89, *p* = 0.0001] than OTUs that decreased, on average (Figures S1, S2). These results, based on sorting OTUs by % change in relative abundance (control vs. treatment), dividing this sorted list into four equal sequential groups (quartiles), and then comparing trait values of the highest (“increasers”) and lowest (“decreasers”) groups, were robust to this group number choice, with all *p* < 0.10 when we divided the sorted list into 3–30 groups (results not shown).

### Associations among soil properties and community-weighted estimated trait means

**Figure 4:**
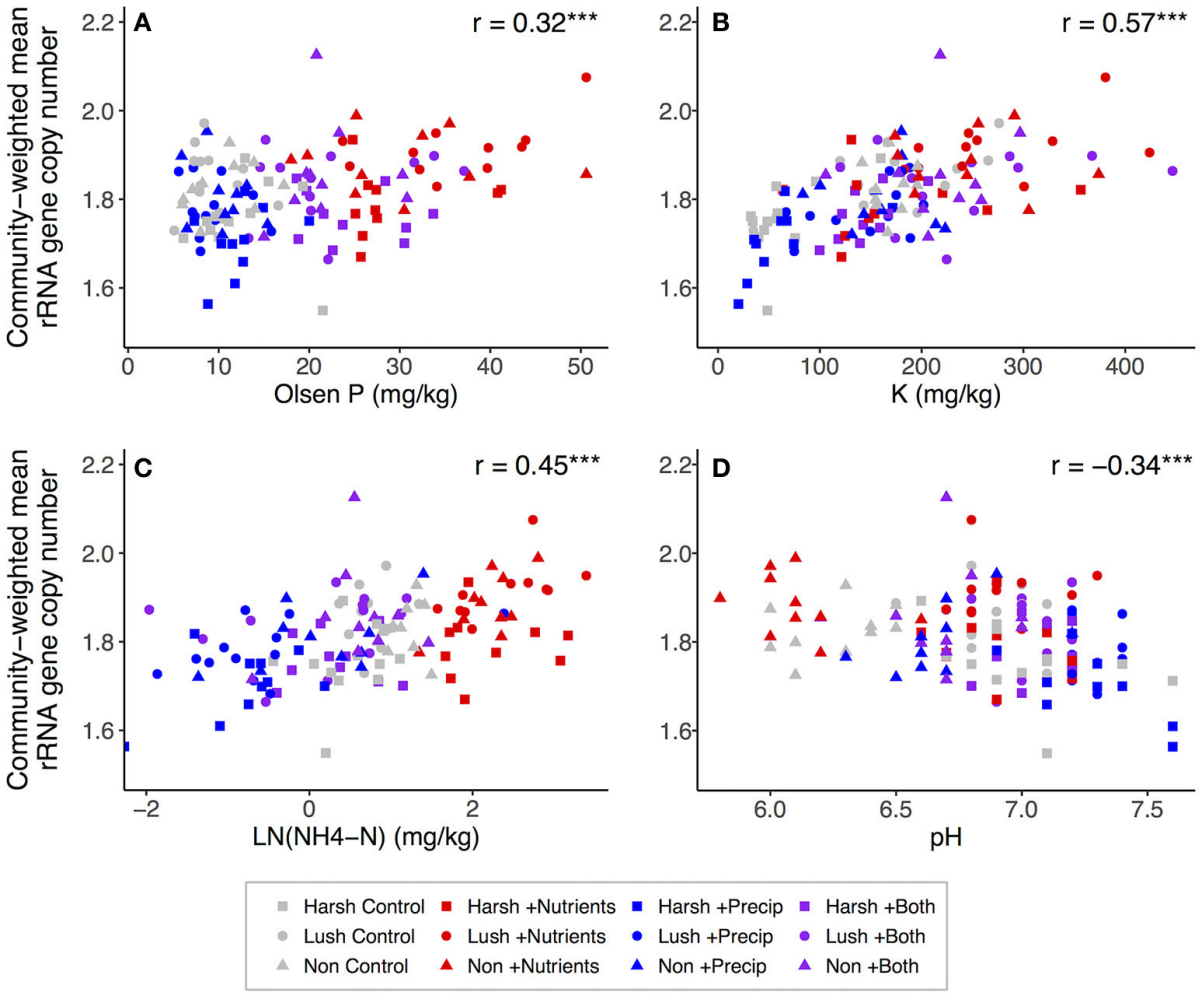
Correlations between selected soil properties and community-weighted mean estimated rRNA gene copy number across all plots (*n* = 129): **(A)** Olsen P, **(B)** K, **(C)** ${\text{NH}}_{4}^{+}$-N, **(D)** pH. ${\text{NH}}_{4}^{+}$-N was log-transformed to better visualize relationships. For correlations: ^*^p < 0.05; ^**^p < 0.01; ^***^p < 0.001.

**Figure 5:**
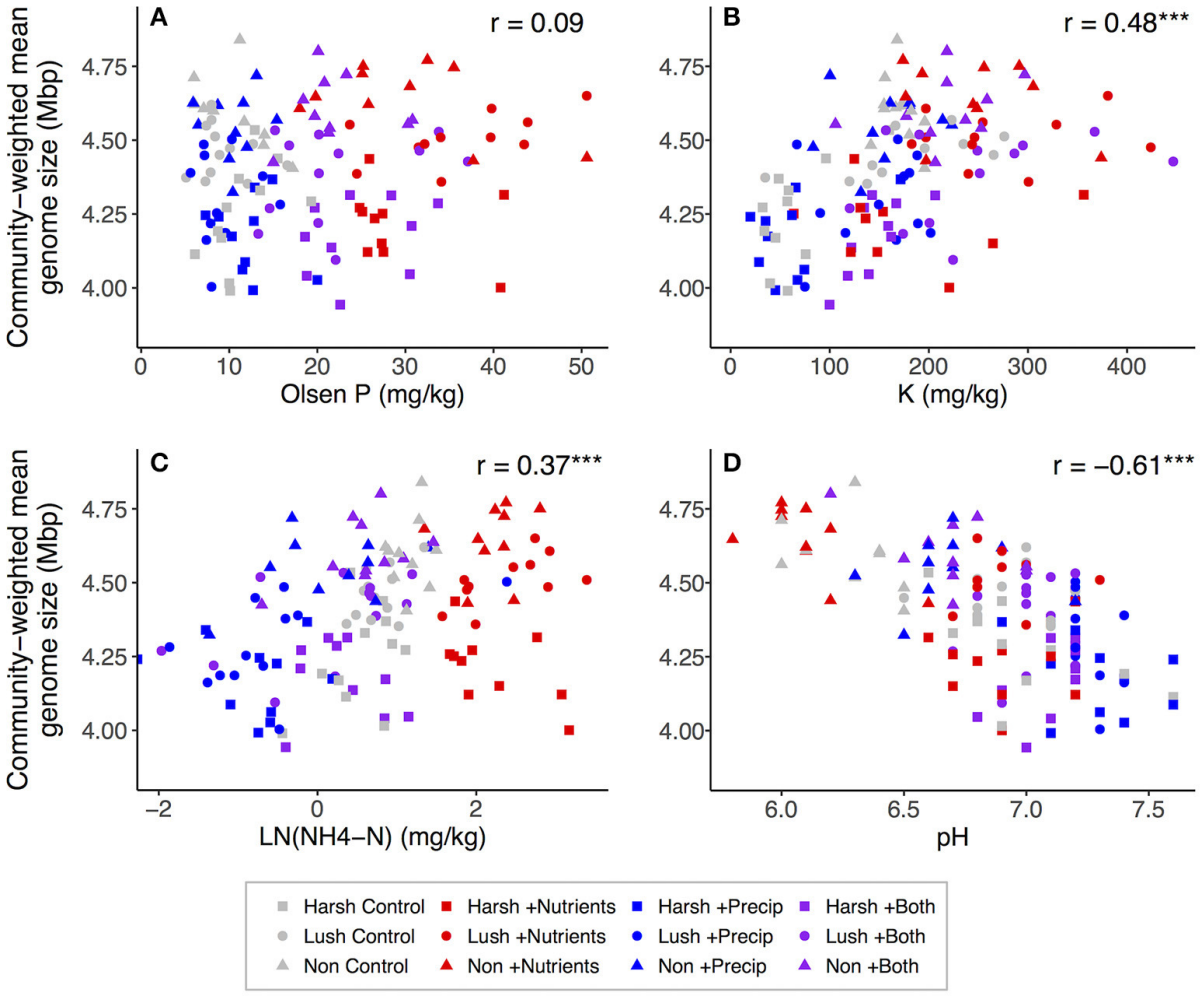
Correlations between selected soil properties and community-weighted mean estimated genome size across all plots (*n* = 129): **(A)** Olsen P, **(B)** K, **(C)** ${\text{NH}}_{4}^{+}$-N, **(D)** pH. ${\text{NH}}_{4}^{+}$-N was log-transformed to better visualize relationships. For correlations: ^*^p < 0.05; ^**^p < 0.01; ^***^ *p* < 0.001.

Both nutrient and precipitation addition remained significant predictors of community-weighted estimated trait means when the majority of measured soil properties were (individually) added to the lme models as covariates (data not shown). However, including ammonium, P, or K as a covariate made the effects of nutrient addition on community-weighted estimated trait means non-significant (*p* > 0.10) for both traits, and for estimated genome size, adding organic matter, DOC, or pH to the model also rendered the nutrient addition effect non-significant (Tables S4, S5). Adding ammonium, extractable N:P, or DOC to the models made the effects of precipitation addition on both community-weighted estimated trait means non-significant (*p* > 0.10), and for estimated genome size, adding pH to the model also rendered the precipitation addition effect non-significant (Tables S4, S5). Considering the correlations between soil properties and community-weighted trait means across all plots, community-weighted mean rRNA gene copy number was significantly positively correlated with CEC, K, organic matter, ammonium, Mg, Ca, P, and extractable N:P and was significantly negatively correlated with pH (Figure 4). Community-weighted mean genome size was significantly positively correlated with Ca, organic matter, CEC, Ca:Mg, K, ammonium, and extractable N:P and was significantly negatively correlated with pH; it was not strongly related to P (Figure 5).

### Effects of soils and treatments on phylum composition and trait changes within phyla

In their distribution of relative abundance across plots, most (23 of 36) phyla showed a soil type preference (Table 1). Relative abundance of approximately half of the phyla changed in response to each of the treatments (Table 1). Treatment response also showed some association with soil type preference: in response to nutrient addition, phyla preferring harsh serpentine tended to decrease and phyla preferring lush and non-serpentine tended to increase, whereas in response to precipitation addition, phyla preferring harsh serpentine tended to increase while those preferring non-serpentine tended to decrease (Table 1). After filtering to exclude very rare phyla (those with ≤0.01% relative abundance across all plots), phyla that increased in response to nutrient addition tended to be those with larger mean estimated genome sizes (*r* = 0.35, *p* = 0.057) but not necessarily greater mean estimated copy numbers (*r* = 0.07, *p* = 0.726), whereas phyla that increased in response to precipitation addition tended to be those with lower mean estimated rRNA gene copy numbers (*r* = −0.33, *p* = 0.066) and slightly smaller mean estimated genome sizes (*r* = −0.29, *p* = 0.109) (Table S6). Considering community-weighted mean estimated trait changes within individual phyla, some phyla exhibited significant changes commensurate with the total community change or there was no significant change, but others showed significant changes in the opposite direction (Table 2).

## Discussion

By building new reference trees of fully sequenced microbial genomes with known trait values, we found that rRNA gene copy number and genome size were phylogenetically conserved and that trait values for unknown taxa could be reliably estimated. Using these estimates to calculate community-weighted mean estimated trait values for soil communities from a grassland environmental change experiment, we found that soil type, nutrient addition, and late-season precipitation addition all significantly shifted the relative abundances of microbes with particular estimated rRNA gene copy number and genome size values within communities. This approach offers promise for improving prediction of microbial environmental change responses.

### Community-weighted mean trait responses to soil type and nutrient addition

The proportion of microbes with many estimated rRNA gene copies was higher in the two more fertile soil types, which had more soil organic matter in addition to higher levels of some inorganic nutrients (Eskelinen and Harrison, 2014), and in the nutrient addition plots, where nutrient levels were elevated (Table S4, Figure 4). OTUs that increased in response to nutrient addition also had higher estimated rRNA gene copy numbers, on average, than OTUs that decreased. Ammonium, P, and K levels were all positively associated with estimated rRNA gene copy number (as well as correlated with one another), suggesting that higher levels of one or more of these nutrients may favor microbes with more rRNA gene copy numbers; however, a role for other nutrient-mediated effects—such as increased plant biomass (Eskelinen and Harrison, 2015c)—cannot be ruled out. Overall, our results are in line with other studies showing that resource-rich conditions are likely to favor “copiotrophic” microbes with higher potential growth rates, which are often correlated with higher rRNA gene copy numbers (Fierer et al., 2007; Vieira-Silva and Rocha, 2010; Roller et al., 2016). In addition to high overall resource levels, high rRNA gene copy numbers tend to be particularly associated with highly pulsed resources (Klappenbach et al., 2000). While we did not collect data on how nutrient addition affected temporal patterns of resource availability for microbes, investigation of that relationship would be a valuable next step for understanding microbial ecological strategies.

Relatively more microbes also had larger estimated genome sizes in the two more fertile soil types and marginally in nutrient addition plots, and OTUs that increased in response to nutrient addition had larger estimated genome sizes, on average, than OTUs that decreased. Higher N availability—both naturally-occurring and resulting from our nutrient addition (Table S4, Figure 5)—was associated with larger estimated genome size, suggesting that it may have favored the retention of additional genes by easing constraints on replication (Giovannoni et al., 2014). In addition to nutrient levels, fluctuations in oxygen supply and/or in the quantity and types of substrates delivered to individual microbes—which tend to favor generalists with larger genomes (Vieira-Silva and Rocha, 2010; Guieysse and Wuertz, 2012; Barberán et al., 2014; Fierer et al., 2014; Giovannoni et al., 2014; Krause et al., 2014)—may have been greater in resource-rich plots due to factors, such as greater root biomass and soil aggregation (Huenneke et al., 1990; Six et al., 2004; Dukes et al., 2005; Riggs et al., 2015; Bach and Hofmockel, 2016; but see Eviner and Chapin, 2002). Conversely, since the lush serpentine and non-serpentine soils have similar nutrient levels and plant biomass (Eskelinen and Harrison, 2015c), the difference in estimated genome size between those soils was likely driven by their differences in other soil chemical properties, such as pH or Ca:Mg (both strongly correlated with estimated genome size).

Interestingly, a recent metagenomic analysis of six grassland N + P addition experiments found a result opposite to ours: several years of N + P addition (at levels similar to those in our experiment) decreased the proportion of microbes with large genomes (Leff et al., 2015). This difference may derive from the different methods used to estimate genome sizes and/or from ecological differences between the study sites. Analysis of their published data combined with data from our three soil types suggests that the effect of N + P addition on community-weighted mean genome size may vary with site pH and/or mean annual precipitation (% genome size change from control vs. pH: *r* = 0.86, *p* = 0.013; vs. mean annual precipitation: *r* = −0.74, *p* = 0.022). However, further study would be needed to test this hypothesis given the small number of sites in the combined data set and the difference in trait estimation methods.

### Community-weighted mean trait responses to precipitation addition

In both the community-weighted mean and increaser vs. decreaser analysis, we found that microbes with fewer estimated rRNA gene copies were favored by late-season precipitation addition, which could relate to their ability to thrive in resource-poor conditions (Fierer et al., 2007; Roller et al., 2016, our data). In our experiment, levels of ammonium were especially decreased by late-season precipitation (Table S4) and could account for the precipitation effect as a covariate (Table S5), suggesting that lower ammonium levels in precipitation addition plots may have contributed to selection for low copy number taxa. Nutrient loss in precipitation addition plots may have resulted from several mechanisms, including greater leaching to below our sampling zone, increased uptake by plants (especially in the plots also receiving nutrient addition, where plant biomass was highest Eskelinen and Harrison, 2015c), and higher nitrification (Gravuer, 2016) and denitrification rates.

The one California grassland study that measured precipitation's effect on microbes with high vs. low rRNA gene copy numbers found the opposite result to ours: measurements 0–72 h after rewetting summer-dry soils revealed increases in relative activity of several high rRNA gene copy number groups, apparently stimulated by the precipitation-mediated resource pulse (Placella et al., 2012). However, our samples were taken several days after a series of weekly precipitation events and suggest that precipitation can favor ecologically different groups of microbes at the end of a wet season than at its onset (Cruz-Martínez et al., 2009, 2012). Our results also likely reflect microbial responses to multiple biotic and abiotic changes accumulated over 3 years of field precipitation manipulations (such as changes in plant composition and productivity) (Li et al., 2017), responses which can substantially differ from those to short-term moisture additions (Evans et al., 2014). These considerations suggest an important caveat when evaluating how the trait responses to precipitation can be generalized: because we measured only a single time point and microbial community responses to precipitation are known to be temporally variable, further studies with temporal sampling will be needed to fully illuminate the temporal pattern of precipitation effects on these microbial traits.

As for rRNA gene copy number, the effect of precipitation addition on community-weighted mean estimated genome size—and the estimated genome sizes of increasers vs. decreasers—were opposite to that of nutrient addition. Ammonium levels, lowest in watered plots, were positively correlated with genome size, suggesting that lower available N levels in watered plots may have favored microbes with more streamlined genomes (Giovannoni et al., 2014). Soil pH was also higher in watered plots and associated with smaller estimated genomes. In addition, root biomass and soil aggregation may have decreased with precipitation addition (Huenneke et al., 1990; Dukes et al., 2005; Chenu and Cosentino, 2011; Bach and Hofmockel, 2015), thus potentially decreasing soil habitat complexity and favoring microbes with smaller genomes, opposite to their influence in nutrient addition plots postulated above. Finally, microbes that thrive at higher soil moisture contents may have narrower moisture niches, lacking genes for the production of exopolymeric substances (EPS) and other mechanisms of drought tolerance (Lennon et al., 2012) and thus possessing smaller genomes. The precipitation addition plots on the high water holding capacity lush serpentine soil may have been especially favorable for microbes with poor drought tolerance, possibly contributing to the stronger precipitation treatment effect on estimated genome size in that soil.

### Relationship between rRNA gene copy number and genome size

We found a moderate correlation between estimated rRNA gene copy number and estimated genome size at the OTU level (*r* = 0.31), suggesting that conditions favoring high rRNA gene copy number might also favor large genome size. Drawing on genome streamlining ideas (Giovannoni et al., 2014), Roller et al. (2016) suggested that high rRNA gene copy numbers and large genomes are part of a characteristic suite of adaptations to high resource conditions, whereas low rRNA gene copy numbers and small genomes characterize microbes adapted to low resource conditions, as also observed by Lauro et al. (2009). While our findings support this idea, we note that other dimensions of the microbial niche—such as temporal pattern of resource delivery, environmental variability, and resource complexity—may at least somewhat independently affect the relative advantage of high or low values of these traits. For example, low rRNA gene copy number with large genome size appears to be favored under some soil conditions (Barberán et al., 2014), such as those with few labile substrates (DeAngelis et al., 2015). Other soil conditions, such as N and P additions to some grassland soils, appear to favor high rRNA gene copy number and small genome size (Leff et al., 2015). Understanding the conditions and microhabitats that favor particular rRNA gene copy number—genome size combinations could advance the definition of ecological strategies for soil microbes.

### Phylogenetic patterns in trait responses

Trait estimation provided a window into the diversity of responses among phyla that would have been difficult to appreciate with composition data alone. There were several phyla within which estimated trait values changed in the opposite direction to that of the community mean. This may be because microbes in different soil microhabitats can respond differently to fertilizer addition and to wet-dry cycles (Ranjard and Richaume, 2001; Neumann et al., 2013), and certain soil microhabitats tend to harbor certain phylogenetic groups (Ruamps et al., 2011; Davinic et al., 2012; Nadeem et al., 2013; Shi et al., 2015; Nuccio et al., 2016). In addition, phyla that tend to occur in densely populated microhabitats, such as the rhizosphere, may experience indirect effects of environmental change via their cooperators' or competitors' responses, which may have smaller effects on phyla that tend to inhabit sparsely populated microhabitats. Additionally, the degree to which trait values in a particular phylum are able to respond to change may depend on the degree of effective variation in that trait within the phylum. Like previous studies in California grasslands (Gutknecht et al., 2012; Matulich et al., 2015), we found substantial independence in the phyla that responded to nutrient vs. precipitation addition, suggesting that different life strategies may be favored by the addition of these two resources as opposed to a simple division between “responders” and “non-responders” (Barnard et al., 2013). Overall, although this variation among phyla indicates that microbial responses were not monolithic, the relative consistency of community-weighted mean estimated trait shifts that we observed across the different soil types suggests that the community-level trait shifts favored by a particular environmental change may ultimately be predictable.

### Utility of trait estimation method

We found correlations between observed and estimated trait values that compare favorably to previous studies and to simulations of likely results from microbial trait estimation procedures (Goberna and Verdú, 2016), likely due to our intentional selection of traits with relatively strong phylogenetic signals and our use of reference trees containing thousands of taxa. Summarizing traits with community-weighted means for DNA-based soil microbial samples almost certainly led to conservative estimates of treatment effect sizes, as DNA from dead and dormant microbes could create inertia masking some of the true change in response to treatments (Lennon and Jones, 2011; Barnard et al., 2014; Carini et al., 2016). Thus, while the effect sizes of soils and treatments on community-weighted mean estimated trait values were small in this study, especially within soils (Figures S3, S4), we believe they are likely to be ecologically relevant. Nevertheless, it will be important to continually evaluate prediction of microbial traits, such as growth strategies and resource specialization as additional fully-sequenced genomes from currently uncultured groups become available, such as genomes from the recently-identified candidate phyla radiation (Brown et al., 2015). Better insight into the ecology of these groups will undoubtedly improve our understanding of complex soil community responses.

Trait estimation provided complementary insight to related approaches. The approach of assigning of “copiotrophic” or “oligotrophic” strategies to phyla and other large taxonomic groups (e.g., Fierer et al., 2007) lends insight into our soil type and nutrient results, as groups previously suggested to be copiotrophs did increase in relative abundance in nutrient-rich plots. However, the relative abundance of these phyla did not respond similarly to the addition of a different resource—water—highlighting the need for complementary approaches to understand responses to some types of environmental change. Relative to summaries of gene abundances from metagenomic data sets, traits may provide a means of synthesizing across gene categories to form a more coherent ecological picture (e.g., Lauro et al., 2009; Le Roux et al., 2016) and may prove more useful than individual metabolic pathways for incorporation into biogeochemical models. Ultimately, simultaneous estimation of gene abundances and trait distributions (as outlined in Fierer et al., 2014) may provide the richest picture. Until more metagenomic data sets accumulate, however, estimation of traits hypothesized to be ecologically important from publically available 16S rRNA data sets could illuminate key patterns, as well as help to target metagenomic analysis.

## Conclusions

In our grassland environmental change experiment, phylogenetic estimation of two traits with hypothesized ecological importance provided a powerful currency with which to compare microbial responses across treatments, soils, and clades (phyla). This approach revealed that two resource additions (water and nutrients)—both of which spurred increases in plant biomass (Eskelinen and Harrison, 2015c)—favored ecologically distinct groups of microbes: while nutrient addition favored potentially faster-growing and more generalist taxa, precipitation addition favored potentially slower-growing and more specialized groups. These relationships may help to refine biogeochemical models that include microbial strategies (e.g., Wieder et al., 2015; Pagel et al., 2016).

As has been found in plants, continued work on microbial traits may reveal that a relatively small number of ecologically important traits capture much of the variation in microbial environmental change responses, and that these traits are, at least in some cases, related to effect traits with impact on ecosystem function (Westoby and Wright, 2006; Lavorel et al., 2007). Exploring linkages between the response traits we identified and effect traits (e.g., Treseder and Lennon, 2015; Amend et al., 2016; Lennon and Lehmkuhl, 2016) could significantly improve our ability to predict changes in ecosystem functioning under global changes (Lavorel and Garnier, 2002; Suding et al., 2008). Predicting the behavior of highly complex microbial communities will likely always retain an element of challenge, but trait-based frameworks are a promising tool for leveraging our vast and growing microbial data bank to pursue this goal.

## Author contributions

AE designed, established and maintained the field experiment; KG designed microbial and soil sampling; KG developed and implemented trait estimation procedure; KG analyzed and interpreted sequence data; KG, AE wrote paper.

### Conflict of interest statement

The authors declare that the research was conducted in the absence of any commercial or financial relationships that could be construed as a potential conflict of interest.
