# Supplementary material for: Nutrient and Rainfall Additions Shift Phylogenetically Estimated Traits of Soil Microbial Communities
Source: Front Microbiol. 2017 Jul 11;8:1271. doi: 10.3389/fmicb.2017.01271 (PMC5504382; doi:10.3389/fmicb.2017.01271)
Supplement: Supplementary file 1 [file DataSheet1.DOCX]

Supplementary Material

Nutrient and rainfall additions shift phylogenetically estimated traits of soil microbial communities

Kelly Gravuer*, Anu Eskelinen

*** Correspondence:** Kelly Gravuer: Kelly.Gravuer@asu.edu

# Supplementary Data

**1.1 Reference tree construction**

The microbial genome reference tree from Kembel et al. (2012) was updated to incorporate genomes that had been sequenced in the years since that study was conducted, including additional genomes from common soil phyla poorly-represented in the original tree (e.g., Acidobacteria, Verrucomicrobia, Planctomycetes). 16S rRNA gene sequences and annotated values for traits of potential ecological importance (Fierer et al., 2014; Krause et al., 2014; Lauro et al., 2009; Madigan et al., 2010) were downloaded for bacterial and archaeal genomes in the Joint Genome Institute’s Integrated Microbial Genomes (IMG) database (Markowitz et al., 2012), for which genome annotation procedures are described by Huntemann et al. (2015), on December 18, 2015. 16S sequences were reduced to one per genome by prioritizing sequences close to the expected length of the gene (1500-1550 bp) and selecting at random among those when more than one was available.

Genomes that have been mostly but not completely assembled (Draft and Permanent Draft genomes in IMG) typically produce estimates of gene presence and functionality that are close to those from completely assembled genomes (Finished genomes in IMG) (Mavromatis et al., 2012). As such, including these genomes in the reference tree appeared desirable, as the likely increase in estimation accuracy due to having more reference taxa, especially from poorly-represented phyla, would likely outweigh any decrease in estimation accuracy that might result from inexact trait value annotations for these taxa. However, functionalities based on counts of gene copies are an important exception. Because these copies represent repeated sequences, they can cause difficulty in ordering and linking scaffolds and are thus likely to be miscounted in Draft and Permanent Draft assemblies. Two separate reference trees were therefore constructed: one that was restricted to high quality, Finished genomes with 10 or fewer scaffolds (“narrow tree”; total taxa = 2330) and one that included genomes with 200 or fewer scaffolds and included Draft and Permanent Draft, as well as Finished, genomes (“broad tree”; total taxa = 9647). The upper limit of 200 scaffolds was set so that the average number of scaffolds in the broad tree was within the range examined by Mavromatis et al. (2012).

Once genomes eligible for inclusion in each of the reference trees were identified, their corresponding 16S rRNA sequences were aligned using PyNAST (Caporaso et al., 2010a) in QIIME 1.9.1 (Caporaso et al., 2010b) and trees were constructed with RAxML 8.2.4 (Stamatakis, 2014) using a GAMMA model of rate heterogeneity.

**1.2 Trait selection and reference tree evaluation**

Candidate traits were selected by screening IMG genome annotations for traits believed to be important to microbial ecology (Fierer et al., 2014; Krause et al., 2014; Lauro et al., 2009; Madigan et al., 2010). To ensure sufficient reference data, only traits annotated for ≥ 35% of genomes in the broad tree (equivalent to ≥ 63% of genomes in the narrow tree) were retained. Candidate traits retained included rRNA gene copy number, genome size, oxygen requirement, and motility. In IMG, oxygen requirement and motility are represented by one of several text descriptors. For analysis and estimation, oxygen requirement terms “Obligate anaerobe” and “Anaerobe” were assigned a value of 0 and terms “Facultative,” “Microaerophilic,” “Aerobe,” and “Obligate aerobe” were assigned a value of 1. This system thus separates taxa known to have only anaerobic metabolism from those capable of aerobic metabolism. For motility, “Non-motile” taxa were coded as 0 and “Motile” and “Chemotactic” taxa were coded as 1.

To assess phylogenetic signal for continuous traits (rRNA gene copy number and genome size), hypothesis tests were conducted using both Pagel’s λ (Pagel, 1999) and Blomberg et al.’s *K* (Blomberg et al., 2003) with the phylosig function of the phytools package (Revell, 2012). Pagel’s λ is a scaling parameter for the observed correlation between species’ trait values on a phylogeny, relative to the correlation expected if the trait value pattern had been produced by Brownian evolution. λ = 0 indicates no correlation between species (i.e., trait value pattern is random) and λ = 1 indicates that the correlation between species is equal to that expected under Brownian evolution (Pagel, 1999). The test of the hypothesis λ = 0 is a likelihood ratio test. Blomberg et al.’s *K* is a ratio of the total variance in trait values among species to the variance that is not explained by the observed phylogenetic relationships. This ratio is then scaled by its expected value under Brownian evolution to allow comparability across different data sets. As for λ, *K* = 1 if the trait value pattern is consistent with Brownian evolution and *K* < 1 if related species are less similar to each other than expected under Brownian evolution (Blomberg et al., 2003). The test of the hypothesis that trait values are random with respect to phylogeny is a randomization test comparing the observed tree to a suite of simulated trees in which trait values are randomly shuffled among tips (Revell, 2012); it was run here with 1000 simulations.

To assess phylogenetic signal for binary traits (oxygen requirement and motility), hypothesis tests were conducted using the *D* statistic (Fritz and Purvis, 2010) as calculated by the phylo.d function (with 1000 permutations) of the caper package (Orme et al., 2013). Fritz and Purvis’s *D* is based on the sum of sister-clade differences across the whole tree. This sum is scaled by both the expected differences if the trait had evolved under Brownian evolution and the expected differences if the trait was random with respect to phylogeny, with both expectations calculated using simulations with the observed trait distribution and tree structure (Fritz and Purvis, 2010). *D* is equal to 1 or higher if the trait value pattern is consistent with a random distribution across the tree tips (i.e., no phylogenetic signal) and to 0 or lower if the pattern is consistent with Brownian evolution (i.e., strong phylogenetic signal) (Fritz and Purvis, 2010). Statistical tests of the hypotheses that *D* = 1 and *D* = 0 are conducted using these two simulated distributions (Orme et al., 2013). Traits between 0 and 1 for which the hypotheses of *D* = 0 and *D* = 1 are both rejected have weak but not statistically significant phylogenetic signal, according to this metric.

To assess trait estimation accuracy, two methods were employed. First, leave-one-out cross-validation was used (as in Kembel et al., 2012), in which species were removed from each reference tree one at a time and their trait values were then estimated as if they were an unknown species. However, because the majority of sequenced microbial genomes are from a few generally easy to culture phyla, leave-one-out cross-validation may suggest better estimation accuracy than would ultimately occur for soil communities. This is because the majority of reference taxa would be found in regions of the tree with many close neighbors, whereas a substantial proportion of soil taxa could be found in regions with few close neighbors. To account for this, a set of 100 “test species” was selected from among the reference species. Test species (listed in Table S3) were selected so that the taxonomic composition of the test set (at the phylum, class, order and family level) was as close as possible to the taxonomic composition of the soil communities from our field experiment. The entire test set was then removed from each reference tree and their trait values were estimated as if they were unknown species. Specifically, the test species were placed onto the reference tree using pplacer (Matsen et al., 2010), and ancestral state estimation methods were used to estimate their trait values based on their phylogenetic positions in relation to reference taxa (via picante:phyEstimate for continuous traits and picante:phyEstimateDisc for binary traits (Kembel et al., 2010)). Only traits that had strong phylogenetic signal and high estimation accuracy as assessed by both the cross-validation and test species methods were retained for further analysis.

Once values for these traits had been estimated for our field experiment samples (using the same pplacer and picante:phyEstimate methods outlined for the test species above) and community-weighted means calculated, we also assessed the degree to which uncertain estimates could potentially lead to spurious results. To do this, community weighted mean trait values were re-calculated excluding the least certain 20% of estimates (OTUs), and ecological conclusions reached with the full vs. restricted OTU set were compared.

**1.3 Prediction accuracy for trait values**

The updated reference trees had substantially more taxa than the originally published tree (2330 and 9647 taxa for the narrow and broad trees, respectively, vs. 484 taxa in original), including more taxa for common but poorly known soil phyla (Table S1). The narrow reference tree performed better for rRNA gene copy number and oxygen requirement, while the broad reference tree performed better for genome size and motility. The following statistics were calculated using the better-performing tree for each trait.

Both continuous traits (rRNA gene copy number and genome size) had significant phylogenetic signal as measured by the *K* and λ metrics. Specifically, rRNA gene copy number had *K* = 0.0012 (*p* = 0.001) and λ = 0.974 (*p* < 0.001), while genome size had *K* = 0.0008 (*p* = 0.001) and λ = 0.994 (*p* < 0.001). Both binary traits also had significant phylogenetic signal, as measured by rejection of the hypothesis that *D* = 1 (i.e., that the trait value pattern was random) in favor of a more phylogenetically clustered pattern (*p* < 0.001 in both cases). However, oxygen requirement (*D* = - 0.006) had a stronger phylogenetic signal than motility (*D* = 0.159); the hypothesis that *D* = 0 (i.e., that the trait value pattern resulted from a Brownian model of evolution) was rejected for motility (*p* < 0.001) but could not be rejected for oxygen requirement (*p* = 0.491). Thus, the phylogenetic clustering of oxygen requirement was as strong as if it had resulted from Brownian evolution, while the clustering for motility was significantly weaker than a Brownian evolution pattern.

The statistical significance of the *K* values for rRNA gene copy number and genome size may seem surprising given their low magnitude. However, as Goberna and Verdú (2016) point out, most prior work with the *K* statistic has involved much narrower taxonomic groups than the two domains of life represented by bacteria and archaea, such that our interpretation of “high” and “low” *K* values may require adjustment at this broad phylogenetic scale. Simulations conducted by these authors also revealed that the number of taxa on the reference tree and their phylogenetic proximity to the taxa to be estimated are also important factors in determining estimate accuracy. In line with this finding, our update of the Kembel et al. (2012) reference tree substantially improved the correlation between actual and estimated test taxa rRNA gene copy number values from 0.79 to 0.89. In fact, our narrow and broad reference trees allowed us to test the relative importance of overall phylogenetic signal vs. a greater number of more closely related reference taxa for estimate accuracy: for genome size, the *K* value for the narrow tree was 19 times larger than that for the broad tree, but the addition of many more genomes in common soil phyla resulted in better prediction accuracy for the broad tree, particularly with the test taxa procedure.

The two methods for testing trait estimation accuracy (leave-one-out cross-validation and the set of 100 test taxa) gave similar results, with the cross-validation method typically reporting slightly higher accuracy. For rRNA gene copy number (which ranges from 1-15 copies), estimates differed from observed values by 0.52 - 0.66 gene copies on average, and for genome size (which ranges from 0.11 - 13.6 Mbp), estimates differed by 0.37 - 0.56 Mbp on average. For both of these traits, estimated values were also highly correlated with actual values (*r* = 0.87 - 0.90 for copy number and 0.90 - 0.92 for genome size). For oxygen requirement, 94% of taxa were correctly classified. Outcomes for these three traits compare favorably to other trait estimation studies and to simulations of possible results from microbial trait estimation procedures (Goberna and Verdú, 2016), likely due to our intentional selection of traits with strong phylogenetic signals and the use of reference trees containing thousands of taxa. However, for motility, only 80 - 88% of taxa were correctly classified. At this point, we decided to remove motility from consideration based on these classification results.

For the remaining three traits, the estimation procedure appeared to work equally well for abundant and rare OTUs in our data set, as evidenced by poor ability of OTU relative abundance to explain trait estimates’ standard errors; statistical models of this relationship had *R*^2^ = 0.0009 for rRNA gene copy number, *R*^2^ = 0.003 for genome size, and *R*^2^ = 0.00002 for oxygen requirement. Comparing model results for community-weighted means calculated using the full set of OTUs vs. for those calculated using only the 80% of OTUs with the most certain trait estimates, the two methods had very similar results for rRNA gene copy number and genome size, increasing confidence that model fits were not driven by the least certain trait estimates (Table S2A-B). Community-weighted means calculated including vs. excluding the least certain estimates for each trait were also correlated at *r* = 0.997 for both copy number and genome size. However, for oxygen requirement, model results diverged between the two methods (Table S2C), and the correlation between the two sets of estimates was only *r* = 0.32. In light of these findings, we decided to exclude oxygen requirement from consideration. In summary, from our suite of testing procedures, we concluded that phylogenetic trait estimation was robust for rRNA gene copy number and genome size, but not sufficiently robust for motility or oxygen requirement.

# Supplementary Tables

**Table S1.** Counts of species by phyla in Kembel et al. (2012), narrow and broad reference trees. Phyla are sorted in decreasing order of total relative abundance in the field experiment data set.

| **Phylum % relative abundance in field experiment** | **Phylum (broken out by class for Proteobacteria)** | **Taxa in original Kembel et al. (2012) reference tree** | **Taxa in narrow reference tree (this study)** | **Taxa in broad reference tree (this study)** |
| --- | --- | --- | --- | --- |
| 22.29 | Actinobacteria | 50 | 232 | 1179 |
| 16.92 | Acidobacteria | 3 | 8 | 26 |
| 11.51 | Verrucomicrobia | 3 | 7 | 23 |
| 10.99 | Alphaproteobacteria | 69 | 210 | 960 |
| 7.60 | Bacteroidetes | 19 | 114 | 806 |
| 5.62 | Betaproteobacteria | 35 | 148 | 542 |
| 5.05 | Gemmatimonadetes | 1 | 2 | 3 |
| 4.72 | Planctomycetes | 1 | 6 | 21 |
| 3.59 | Chloroflexi | 7 | 17 | 34 |
| 2.97 | Deltaproteobacteria | 25 | 59 | 178 |
| 2.71 | Crenarchaeota | 0 | 40 | 77 |
| 2.31 | Gammaproteobacteria | 89 | 501 | 2147 |
| 0.63 | Armatimonadetes | 0 | 2 | 4 |
| 0.49 | Elusimicrobia | 0 | 3 | 3 |
| 0.48 | Chlorobi | 11 | 14 | 18 |
| 0.29 | Nitrospirae | 1 | 6 | 11 |
| 0.21 | Cyanobacteria | 24 | 78 | 178 |
| 0.19 | Unassigned Proteobacteria | 0 | 0 | 19 |
| 0.18 | FBP | 0 | 0 | 0 |
| 0.18 | Tenericutes | 18 | 81 | 178 |
| 0.18 | TM7 | 0 | 2 | 13 |
| 0.12 | TM6 | 0 | 1 | 9 |
| 0.11 | Euryarchaeota | 0 | 97 | 171 |
| 0.10 | Chlamydiae | 5 | 36 | 53 |
| 0.09 | BRC1 | 0 | 0 | 0 |
| 0.09 | Fibrobacteres | 0 | 2 | 4 |
| 0.08 | OD1 | 0 | 0 | 69 |
| 0.08 | WS3 | 0 | 0 | 3 |
| 0.07 | OP3 | 0 | 0 | 4 |
| 0.05 | Firmicutes | 83 | 464 | 2240 |
| 0.04 | [Parvarchaeota] | 0 | 0 | 4 |
| 0.01 | WPS-2 | 0 | 0 | 0 |
| 0.01 | OP11 | 0 | 0 | 31 |
| 0.01 | MVP-21 | 0 | 0 | 0 |
| 0.01 | GN02 | 0 | 0 | 10 |
| 0.01 | WS2 | 0 | 0 | 0 |
| 0.005 | BHI80-139 | 0 | 0 | 0 |
| 0.004 | NKB19 | 0 | 0 | 1 |
| 0.002 | SR1 | 0 | 0 | 2 |
| 0.002 | AD3 | 0 | 0 | 0 |
| 0.0007 | Spirochaetes | 7 | 49 | 152 |
| 0.0007 | [Thermi] | 4 | 19 | 56 |
| 0.0003 | WS4 | 0 | 0 | 0 |
| 0.0002 | ZB3 | 0 | 0 | 0 |
| 0.0001 | Fusobacteria | 1 | 13 | 42 |
| 0.00006 | SBR1093 | 0 | 0 | 0 |
| 0.00003 | Kazan-3B-28 | 0 | 0 | 0 |
| 0.00002 | Epsilonproteobacteria | 15 | 78 | 231 |
| 0 | Aquificae | 5 | 12 | 16 |
| 0 | Caldiserica | 0 | 1 | 1 |
| 0 | Caldithrix | 0 | 0 | 1 |
| 0 | CD12 | 0 | 0 | 2 |
| 0 | Chrysiogenetes | 0 | 1 | 2 |
| 0 | Deferribacteres | 0 | 4 | 7 |
| 0 | Dictyoglomi | 1 | 2 | 2 |
| 0 | EM19 | 0 | 0 | 6 |
| 0 | EM3 | 0 | 0 | 2 |
| 0 | KSB3 | 0 | 0 | 1 |
| 0 | Lentisphaerae | 0 | 0 | 3 |
| 0 | Nanoarchaeota | 0 | 0 | 1 |
| 0 | NC10 | 0 | 0 | 2 |
| 0 | OctSpA1-106 | 0 | 0 | 3 |
| 0 | OP1 | 0 | 0 | 2 |
| 0 | OP8 | 0 | 0 | 4 |
| 0 | OP9 | 0 | 0 | 8 |
| 0 | Poribacteria | 0 | 0 | 2 |
| 0 | SAR406 | 0 | 0 | 6 |
| 0 | Synergistetes | 0 | 4 | 20 |
| 0 | Thermotogae | 7 | 17 | 28 |
| 0 | WPS-2 | 0 | 0 | 1 |
| 0 | WS5 | 0 | 0 | 2 |
| 0 | WS6 | 0 | 0 | 3 |
| 0 | WWE1 | 0 | 0 | 7 |
| **100.00** | **TOTAL** | **484** | **2330** | **9647** |

**Table S2.** Side-by-side linear mixed effects model tables testing effects of experimental treatments on community-weighted mean estimated trait values, using two methods for community-weighted mean calculation. One set of means was calculated using trait estimates for all taxa, while the second set of means was calculated after removing the 20% of taxa with the least certain trait value estimates (as determined by standard error) from the experiment-wide data set. Model results were similar between methods for rRNA gene copy number (A) and genome size (B), but for oxygen requirement (C), removal of the least certain 20% of taxa changed inferences about which experimental factors significantly affected this trait. As such, we decided that our estimation method for oxygen requirement was not robust enough to use for making ecological inferences about our data set.

| **A. rRNA gene copy number** |  | **All estimates** | | **Least certain 20% of estimates removed** | |
| --- | --- | --- | --- | --- | --- |
| **Factor** | **df** | ***F*** | ***p*** | ***F*** | ***p*** |
| Soil type | 2, 109 | 17.26 | < 0.0001 | 19.27 | < 0.0001 |
| Precipitation | 1, 109 | 13.54 | 0.0004 | 12.57 | 0.001 |
| Nutrients | 1, 109 | 18.29 | < 0.0001 | 21.25 | < 0.0001 |
| Soil type x Precipitation | 2, 109 | 1.46 | 0.236 | 1.36 | 0.262 |
| Soil type x Nutrients | 2, 109 | 0.15 | 0.864 | 0.08 | 0.928 |
| Precipitation x Nutrients | 1, 109 | 0.003 | 0.958 | 0.01 | 0.946 |
| Soil type x Precipitation x Nutrients | 2, 109 | 0.13 | 0.882 | 0.16 | 0.854 |

| **B. Genome size** |  | **All estimates** | | **Least certain 20% of estimates removed** | |
| --- | --- | --- | --- | --- | --- |
| **Factor** | **df** | ***F*** | ***p*** | ***F*** | ***p*** |
| Soil type | 2, 109 | 70.40 | < 0.0001 | 75.26 | < 0.0001 |
| Precipitation | 1, 109 | 10.85 | 0.001 | 11.37 | 0.001 |
| Nutrients | 1, 109 | 3.32 | 0.071 | 3.20 | 0.076 |
| Soil type x Precipitation | 2, 109 | 2.53 | 0.084 | 2.65 | 0.075 |
| Soil type x Nutrients | 2, 109 | 0.59 | 0.555 | 0.74 | 0.479 |
| Precipitation x Nutrients | 1, 109 | 0.09 | 0.759 | 0.12 | 0.732 |
| Soil type x Precipitation x Nutrients | 2, 109 | 0.15 | 0.860 | 0.18 | 0.838 |

| **C. Oxygen requirement** |  | **All estimates** | | **Least certain 20% of estimates removed** | |
| --- | --- | --- | --- | --- | --- |
| **Factor** | **df** | ***F*** | ***p*** | ***F*** | ***p*** |
| Soil type | 2, 109 | 6.31 | 0.003 | 30.13 | < 0.0001 |
| Precipitation | 1, 109 | 2.92 | 0.090 | 4.76 | 0.031 |
| Nutrients | 1, 109 | 8.21 | 0.005 | 4.87 | 0.029 |
| Soil type x Precipitation | 2, 109 | 1.02 | 0.363 | 1.13 | 0.327 |
| Soil type x Nutrients | 2, 109 | 0.00 | 0.996 | 2.14 | 0.122 |
| Precipitation x Nutrients | 1, 109 | 9.15 | 0.003 | 0.06 | 0.813 |
| Soil type x Precipitation x Nutrients | 2, 109 | 1.19 | 0.308 | 1.82 | 0.167 |

**Table S3A.** List of 100 test species with observed and estimated trait values. Test species were selected from the list of reference taxa such that the taxonomic composition of the test set (at the phylum, class, order and family level) was as close as possible to the taxonomic composition of the soil communities from our field experiment. The entire test set was then removed from each reference tree, their trait values were estimated as if they were unknown species, and the estimates were compared to actual trait values to assess tree performance.

|  | *rRNA gene copy number* | | | *Genome size* | | *Oxygen requirement* | | *Motility* | |
| --- | --- | --- | --- | --- | --- | --- | --- | --- | --- |
| **Species** | **Actual** | **Estimated** | **Estimate with Kembel et al. (2012) ref. tree** | **Actual** | **Estimated** | **Actual** | **Estimated** | **Actual** | **Estimated** |
| *Acidobacterium capsulatum* | 1 | 1 | 2 | 4.13 | 4.95 | 1 | 1 | 0 | 0 |
| *Candidatus Koribacter versatilis* | 1 | 1 | 2 | 5.65 | 5.04 | 1 | 1 | 0 | 0 |
| *Candidatus Solibacter usitatus* | 2 | 1 | 2 | 9.97 | 6.15 | 1 | 1 | 0 | 0 |
| *Ilumatobacter coccineum* | 2 | 2 | 2 | 4.83 | 1.14 | 1 | 1 | 0 | 0 |
| *Kytococcus sedentarius* | 2 | 2 | 3 | 2.79 | 3.55 | 1 | 1 | 0 | 0 |
| *Frankia alni* | 2 | 2 | 2 | 7.50 | 6.80 | 1 | 1 | 0 | 0 |
| *Frankia* sp. | 2 | 2 | 2 | 5.32 | 5.57 | 1 | 1 | 0 | 0 |
| *Kineococcus radiotolerans* | 4 | 2 | 4 | 4.96 | 4.13 | 1 | 1 | 1 | 0 |
| *Rhodoluna lacicola* | 1 | 1 | 1 | 1.43 | 1.19 | 1 | 1 | 0 | 0 |
| *Renibacterium salmoninarum* | 2 | 5 | 5 | 3.16 | 3.75 | 1 | 1 | 0 | 1 |
| *Salinispora arenicola* | 3 | 3 | 3 | 5.79 | 5.87 | 1 | 1 | 1 | 1 |
| *Mycobacterium vanbaalenii* | 2 | 2 | 2 | 6.49 | 6.33 | 1 | 1 | 0 | 0 |
| *Nocardia nova* | 3 | 3 | 3 | 8.35 | 9.54 | 1 | 1 | 1 | 0 |
| *Nocardioides* sp. | 2 | 2 | 3 | 5.29 | 4.56 | 1 | 1 | 0 | 1 |
| *Actinosynnema mirum* | 5 | 4 | 3 | 8.25 | 8.72 | 1 | 1 | 0 | 0 |
| *Saccharomonospora viridis* | 3 | 3 | 4 | 4.31 | 4.64 | 1 | 1 | 0 | 0 |
| *Streptomyces griseus* | 6 | 6 | 6 | 8.55 | 7.89 | 1 | 1 | 0 | 0 |
| *Streptosporangium roseum* | 6 | 4 | 3 | 10.37 | 7.96 | 1 | 1 | 0 | 0 |
| *Rubrobacter xylanophilus* | 1 | 1 | 2 | 3.23 | 3.37 | 1 | 1 | 0 | 0 |
| *Conexibacter woesei* | 1 | 1 | 2 | 6.36 | 6.09 | 1 | 1 | 1 | 0 |
| *Paludibacter propionicigenes* | 3 | 4 | 3 | 3.69 | 2.77 | 0 | 0 | 0 | 0 |
| *Alistipes finegoldii* | 2 | 2 | 3 | 3.73 | 3.47 | 0 | 1 | 0 | 0 |
| *Leadbetterella byssophila* | 3 | 2 | 3 | 4.06 | 5.37 | 1 | 1 | 0 | 0 |
| *Marivirga tractuosa* | 2 | 3 | 2 | 4.52 | 4.91 | 1 | 1 | 1 | 0 |
| *Blattabacterium sp.* | 1 | 1 | 2 | 0.64 | 0.63 | 1 | 1 | 0 | 0 |
| *Owenweeksia hongkongensis* | 2 | 3 | 3 | 4.00 | 4.08 | 1 | 1 | 1 | 0 |
| *Capnocytophaga ochracea* | 4 | 3 | 4 | 2.61 | 2.74 | 1 | 1 | 1 | 1 |
| *Flavobacterium johnsoniae* | 6 | 2 | 5 | 6.10 | 4.17 | 1 | 1 | 1 | 0 |
| *Zunongwangia profunda* | 3 | 3 | 3 | 5.13 | 4.02 | 1 | 1 | 0 | 0 |
| *Chitinophaga pinensis* | 6 | 3 | 3 | 9.13 | 8.47 | 1 | 1 | 1 | 0 |
| *Haliscomenobacter hydrossis* | 2 | 3 | 2 | 8.77 | 7.01 | 1 | 1 | 0 | 1 |
| *Pedobacter saltans* | 4 | 4 | 2 | 4.64 | 5.05 | 1 | 1 | 0 | 0 |
| *Roseiflexus* sp. | 2 | 2 | 2 | 5.80 | 5.03 | 1 | 1 | 1 | 1 |
| *Anabaena* sp. | 5 | 5 | 4 | 5.31 | 5.11 | 1 | 1 | 1 | 1 |
| *Kyrpidia tusciae* | 5 | 6 | 3 | 3.38 | 3.51 | 1 | 1 | 1 | 1 |
| *Geobacillus kaustophilus* | 9 | 8 | 10 | 3.59 | 3.35 | 1 | 1 | 1 | 1 |
| *Lysinibacillus sphaericus* | 10 | 10 | 9 | 4.82 | 4.79 | 1 | 1 | 1 | 1 |
| *Paenibacillus lautus* | 8 | 10 | 11 | 7.12 | 6.95 | 1 | 1 | 1 | 1 |
| *Staphylococcus carnosus* | 5 | 6 | 6 | 2.57 | 2.63 | 1 | 1 | 0 | 0 |
| *Enterococcus casseliflavus* | 5 | 5 | 4 | 3.43 | 3.45 | 1 | 1 | 0 | 1 |
| *Lactobacillus reuteri* | 6 | 6 | 6 | 2.04 | 1.95 | 1 | 1 | 0 | 0 |
| *Clostridium saccharolyticum* | 6 | 4 | 6 | 4.66 | 5.04 | 0 | 0 | 1 | 1 |
| *Clostridium clariflavum* | 6 | 4 | 4 | 4.90 | 4.39 | 0 | 0 | 0 | 1 |
| *Ruminococcus* sp. | 1 | 4 | 5 | 2.57 | 3.97 | 0 | 0 | 0 | 0 |
| *Gemmatimonas aurantiaca* | 1 | 2 | 2 | 4.64 | 4.84 | 1 | 0 | 1 | 1 |
| *Planctomyces brasiliensis* | 2 | 2 | 2 | 6.01 | 6.91 | 1 | 1 | 1 | 1 |
| *Rhodopirellula baltica* | 1 | 2 | 2 | 7.15 | 7.53 | 1 | 1 | 1 | 1 |
| *Asticcacaulis excentricus* | 3 | 2 | 2 | 4.31 | 4.16 | 1 | 1 | 1 | 1 |
| *Rhodopseudomonas palustris* | 2 | 1 | 1 | 4.89 | 5.34 | 1 | 1 | 1 | 1 |
| *Methylobacterium extorquens* | 5 | 5 | 6 | 5.47 | 6.69 | 1 | 1 | 1 | 1 |
| *Mesorhizobium opportunistum* | 2 | 2 | 2 | 6.88 | 6.53 | 1 | 1 | 1 | 1 |
| *Ensifer meliloti* | 3 | 3 | 3 | 6.69 | 6.88 | 1 | 1 | 1 | 1 |
| *Parvibaculum lavamentivorans* | 1 | 2 | 2 | 3.91 | 3.44 | 1 | 1 | 1 | 1 |
| *Hirschia baltica* | 2 | 2 | 2 | 3.54 | 3.36 | 1 | 1 | 1 | 1 |
| *Maricaulis maris* | 2 | 2 | 2 | 3.37 | 3.17 | 1 | 1 | 1 | 1 |
| *Paracoccus denitrificans* | 3 | 4 | 2 | 5.24 | 5.19 | 1 | 1 | 0 | 0 |
| *Rhodobacter capsulatus* | 4 | 4 | 3 | 3.87 | 4.31 | 1 | 1 | 1 | 0 |
| *Gluconacetobacter diazotrophicus* | 4 | 4 | 3 | 3.91 | 3.96 | 1 | 1 | 1 | 1 |
| *Azospirillum sp.* | 9 | 8 | 3 | 7.60 | 7.00 | 1 | 1 | 1 | 1 |
| *Rickettsia bellii* | 1 | 1 | 1 | 1.52 | 1.36 | 1 | 1 | 0 | 0 |
| *Rickettsia typhi* | 1 | 1 | 1 | 1.11 | 1.16 | 1 | 1 | 0 | 0 |
| *Erythrobacter litoralis* | 1 | 1 | 1 | 3.05 | 2.91 | 1 | 1 | 1 | 1 |
| *Novosphingobium aromaticivorans* | 3 | 2 | 1 | 4.23 | 4.76 | 1 | 1 | 0 | 1 |
| *Sphingomonas sanxanigenens* | 3 | 2 | 2 | 6.21 | 3.98 | 1 | 1 | 0 | 1 |
| *Advenella mimigardefordensis* | 2 | 2 | 3 | 4.76 | 4.34 | 1 | 1 | 1 | 0 |
| *Burkholderia xenovorans* | 6 | 6 | 5 | 9.73 | 9.70 | 1 | 1 | 1 | 1 |
| *Polynucleobacter necessarius* | 1 | 1 | 1 | 2.16 | 1.52 | 1 | 1 | 0 | 0 |
| *Acidovorax avenae* | 3 | 2 | 3 | 5.48 | 5.53 | 1 | 1 | 1 | 1 |
| *Variovorax paradoxus* | 2 | 2 | 5 | 6.55 | 7.15 | 1 | 1 | 1 | 1 |
| *Methylotenera mobilis* | 2 | 3 | 2 | 2.55 | 2.64 | 1 | 1 | 1 | 1 |
| *Methylovorus* sp. | 2 | 2 | 2 | 2.86 | 2.67 | 1 | 1 | 1 | 1 |
| *Laribacter hongkongensis* | 7 | 6 | 5 | 3.17 | 3.11 | 0 | 1 | 1 | 0 |
| *Nitrosomonas* sp*.* | 1 | 1 | 1 | 3.34 | 3.50 | 1 | 1 | 1 | 1 |
| *Dechloromonas aromatica* | 4 | 2 | 2 | 4.50 | 3.52 | 1 | 1 | 1 | 1 |
| *Thauera aminoaromatica* | 4 | 4 | 3 | 4.57 | 4.81 | 1 | 1 | 1 | 1 |
| *Bdellovibrio bacteriovorus* | 2 | 1 | 3 | 3.78 | 2.88 | 1 | 1 | 1 | 1 |
| *Geobacter sulfurreducens* | 2 | 3 | 3 | 3.81 | 3.91 | 0 | 0 | 1 | 1 |
| *Haliangium ochraceum* | 2 | 3 | 3 | 9.45 | 9.72 | 1 | 0 | 1 | 1 |
| *Anaeromyxobacter dehalogenans* | 2 | 2 | 2 | 5.01 | 5.03 | 0 | 0 | 1 | 1 |
| *Myxococcus xanthus* | 4 | 3 | 2 | 9.14 | 9.27 | 1 | 1 | 1 | 1 |
| *Syntrophus aciditrophicus* | 1 | 2 | 3 | 3.18 | 3.69 | 0 | 0 | 0 | 1 |
| *Syntrophobacter fumaroxidans* | 2 | 2 | 3 | 4.99 | 3.32 | 0 | 0 | 0 | 1 |
| *Alteromonas* sp. | 5 | 5 | 5 | 4.97 | 4.64 | 1 | 1 | 1 | 1 |
| *Glaciecola nitratireducens* | 4 | 5 | 5 | 4.13 | 4.10 | 1 | 1 | 1 | 1 |
| *Shewanella halifaxensis* | 10 | 11 | 8 | 5.23 | 5.15 | 1 | 1 | 1 | 1 |
| *Allochromatium vinosum* | 3 | 3 | 2 | 3.67 | 5.18 | 0 | 0 | 1 | 1 |
| *Halorhodospira halophila* | 2 | 2 | 2 | 2.68 | 3.24 | 0 | 0 | 1 | 1 |
| *Thioalkalivibrio sulfidophilus* | 1 | 1 | 2 | 3.46 | 3.05 | 1 | 1 | 1 | 1 |
| *Escherichia coli* | 7 | 7 | 7 | 5.11 | 5.02 | 1 | 1 | 1 | 1 |
| *Salmonella bongori* | 7 | 7 | 7 | 4.46 | 4.77 | 1 | 1 | 1 | 1 |
| *Coxiella burnetii* | 1 | 1 | 1 | 2.01 | 2.02 | 1 | 1 | 1 | 1 |
| *Legionella pneumophila* | 3 | 3 | 3 | 3.40 | 3.46 | 1 | 1 | 1 | 1 |
| *Frateuria aurantia* | 4 | 2 | 2 | 3.60 | 4.66 | 1 | 1 | 1 | 1 |
| *Acinetobacter baumannii* | 6 | 6 | 5 | 4.05 | 3.94 | 1 | 1 | 0 | 0 |
| *Acinetobacter baylyi* | 7 | 4 | 5 | 3.60 | 3.61 | 1 | 1 | 0 | 0 |
| *Pseudomonas fluorescens* | 6 | 3 | 4 | 6.44 | 6.37 | 1 | 1 | 1 | 1 |
| *Pseudomonas mendocina* | 4 | 4 | 5 | 5.07 | 5.11 | 1 | 1 | 1 | 1 |
| *Xanthomonas citri* | 2 | 2 | 2 | 5.40 | 5.04 | 1 | 1 | 1 | 1 |
| *Opitutus terrae* | 1 | 2 | 2 | 5.96 | 4.79 | 0 | 1 | 1 | 0 |
| *Akkermansia muciniphila* | 3 | 1 | 2 | 2.66 | 5.13 | 0 | 1 | 0 | 0 |

**Table S3B.** Taxonomic and identifier information from IMG for each test species.

| **IMG Genome ID** | **Phylum** | **Class** | **Order** | **Family** | **Species** |
| --- | --- | --- | --- | --- | --- |
| 643692001 | Acidobacteria | Acidobacteriia | Acidobacteriales | Acidobacteriaceae | *Acidobacterium capsulatum* |
| 637000001 | Acidobacteria | Acidobacteriia | Acidobacteriales | Acidobacteriaceae | *Candidatus Koribacter versatilis* |
| 639633060 | Acidobacteria | Solibacteres | Solibacterales | Solibacteraceae | *Candidatus Solibacter usitatus* |
| 2545824624 | Actinobacteria | Actinobacteria | Acidimicrobiales | Acidimicrobiaceae | *Ilumatobacter coccineum* |
| 644736380 | Actinobacteria | Actinobacteria | Actinomycetales | Dermacoccaceae | *Kytococcus sedentarius* |
| 2623620699 | Actinobacteria | Actinobacteria | Actinomycetales | Frankiaceae | *Frankia alni* |
| 2506783011 | Actinobacteria | Actinobacteria | Actinomycetales | Frankiaceae | *Frankia symbiont* |
| 640753031 | Actinobacteria | Actinobacteria | Actinomycetales | Kineosporiaceae | *Kineococcus radiotolerans* |
| 2504643007 | Actinobacteria | Actinobacteria | Actinomycetales | Microbacteriaceae | *Rhodoluna lacicola* |
| 641228502 | Actinobacteria | Actinobacteria | Actinomycetales | Micrococcaceae | *Renibacterium salmoninarum* |
| 641228504 | Actinobacteria | Actinobacteria | Actinomycetales | Micromonosporaceae | *Salinispora arenicola* |
| 639633044 | Actinobacteria | Actinobacteria | Actinomycetales | Mycobacteriaceae | *Mycobacterium vanbaalenii* |
| 2547132424 | Actinobacteria | Actinobacteria | Actinomycetales | Nocardiaceae | *Nocardia nova* |
| 639633046 | Actinobacteria | Actinobacteria | Actinomycetales | Nocardioidaceae | *Nocardioides sp.* |
| 644736323 | Actinobacteria | Actinobacteria | Actinomycetales | Pseudonocardiaceae | *Actinosynnema mirum* |
| 644736404 | Actinobacteria | Actinobacteria | Actinomycetales | Pseudonocardiaceae | *Saccharomonospora viridis* |
| 641522653 | Actinobacteria | Actinobacteria | Actinomycetales | Streptomycetaceae | *Streptomyces griseus* |
| 646311958 | Actinobacteria | Actinobacteria | Actinomycetales | Streptosporangiaceae | *Streptosporangium roseum* |
| 637000248 | Actinobacteria | Actinobacteria | Rubrobacterales | Rubrobacteraceae | *Rubrobacter xylanophilus* |
| 646311917 | Actinobacteria | Actinobacteria | Solirubrobacterales | Conexibacteraceae | *Conexibacter woesei* |
| 649633080 | Bacteroidetes | Bacteroidia | Bacteroidales | Porphyromonadaceae | *Paludibacter propionicigenes* |
| 2509601035 | Bacteroidetes | Bacteroidia | Bacteroidales | Rikenellaceae | *Alistipes finegoldii* |
| 649633063 | Bacteroidetes | Cytophagia | Cytophagales | Cytophagaceae | *Leadbetterella byssophila* |
| 649633065 | Bacteroidetes | Cytophagia | Cytophagales | Flammeovirgaceae | *Marivirga tractuosa* |
| 650716011 | Bacteroidetes | Flavobacteriia | Flavobacteriales | Blattabacteriaceae | *Blattabacterium sp.* |
| 2508501098 | Bacteroidetes | Flavobacteriia | Flavobacteriales | Cryomorphaceae | *Owenweeksia hongkongensis* |
| 644736338 | Bacteroidetes | Flavobacteriia | Flavobacteriales | Flavobacteriaceae | *Capnocytophaga ochracea* |
| 644736369 | Bacteroidetes | Flavobacteriia | Flavobacteriales | Flavobacteriaceae | *Flavobacterium johnsoniae* |
| 646564591 | Bacteroidetes | Flavobacteriia | Flavobacteriales | Flavobacteriaceae | *Zunongwangia profunda* |
| 644736340 | Bacteroidetes | Sphingobacteriia | Sphingobacteriales | Chitinophagaceae | *Chitinophaga pinensis* |
| 2504756004 | Bacteroidetes | Sphingobacteriia | Sphingobacteriales | Saprospiraceae | *Haliscomenobacter hydrossis* |
| 649633082 | Bacteroidetes | Sphingobacteriia | Sphingobacteriales | Sphingobacteriaceae | *Pedobacter saltans* |
| 640427139 | Chloroflexi | Chloroflexi | Chloroflexales | Chloroflexaceae | *Roseiflexus sp.* |
| 2561511140 | Cyanobacteria | unclassified | Nostocales | Nostocaceae | *Anabaena sp.* |
| 646564511 | Firmicutes | Bacilli | Bacillales | Alicyclobacillaceae | *Kyrpidia tusciae* |
| 2623620701 | Firmicutes | Bacilli | Bacillales | Bacillaceae | *Geobacillus kaustophilus* |
| 641522637 | Firmicutes | Bacilli | Bacillales | Bacillaceae | *Lysinibacillus sphaericus* |
| 646311929 | Firmicutes | Bacilli | Bacillales | Paenibacillaceae | *Paenibacillus lautus* |
| 643692037 | Firmicutes | Bacilli | Bacillales | Staphylococcaceae | *Staphylococcus carnosus* |
| 2540341159 | Firmicutes | Bacilli | Lactobacillales | Enterococcaceae | *Enterococcus casseliflavus* |
| 642555135 | Firmicutes | Bacilli | Lactobacillales | Lactobacillaceae | *Lactobacillus reuteri* |
| 648028018 | Firmicutes | Clostridia | Clostridiales | Clostridiaceae | *Clostridium saccharolyticum* |
| 2507262051 | Firmicutes | Clostridia | Clostridiales | Ruminococcaceae | *Clostridium clariflavum* |
| 650377969 | Firmicutes | Clostridia | Clostridiales | Ruminococcaceae | *Ruminococcus sp.* |
| 643692024 | Gemmatimonadetes | Gemmatimonadetes | Gemmatimonadales | Gemmatimonadaceae | *Gemmatimonas aurantiaca* |
| 649633083 | Planctomycetes | Planctomycetia | Planctomycetales | Planctomycetaceae | *Planctomyces brasiliensis* |
| 2623620638 | Planctomycetes | Planctomycetia | Planctomycetales | Planctomycetaceae | *Rhodopirellula baltica* |
| 649633007 | Proteobacteria | Alphaproteobacteria | Caulobacterales | Caulobacteraceae | *Asticcacaulis excentricus* |
| 637000238 | Proteobacteria | Alphaproteobacteria | Rhizobiales | Bradyrhizobiaceae | *Rhodopseudomonas palustris* |
| 641228497 | Proteobacteria | Alphaproteobacteria | Rhizobiales | Methylobacteriaceae | *Methylobacterium extorquens* |
| 2503198000 | Proteobacteria | Alphaproteobacteria | Rhizobiales | Phyllobacteriaceae | *Mesorhizobium opportunistum* |
| 2623620857 | Proteobacteria | Alphaproteobacteria | Rhizobiales | Rhizobiaceae | *Ensifer meliloti* |
| 640753040 | Proteobacteria | Alphaproteobacteria | Rhizobiales | Rhodobiaceae | *Parvibaculum lavamentivorans* |
| 644736375 | Proteobacteria | Alphaproteobacteria | Rhodobacterales | Hyphomonadaceae | *Hirschia baltica* |
| 637000157 | Proteobacteria | Alphaproteobacteria | Rhodobacterales | Hyphomonadaceae | *Maricaulis maris* |
| 639633048 | Proteobacteria | Alphaproteobacteria | Rhodobacterales | Rhodobacteraceae | *Paracoccus denitrificans* |
| 646564563 | Proteobacteria | Alphaproteobacteria | Rhodobacterales | Rhodobacteraceae | *Rhodobacter capsulatus* |
| 643348555 | Proteobacteria | Alphaproteobacteria | Rhodospirillales | Acetobacteraceae | *Gluconacetobacter diazotrophicus* |
| 646311907 | Proteobacteria | Alphaproteobacteria | Rhodospirillales | Rhodospirillaceae | *Azospirillum sp.* |
| 2623620639 | Proteobacteria | Alphaproteobacteria | Rickettsiales | Rickettsiaceae | *Rickettsia bellii* |
| 2623620644 | Proteobacteria | Alphaproteobacteria | Rickettsiales | Rickettsiaceae | *Rickettsia typhi* |
| 2623620814 | Proteobacteria | Alphaproteobacteria | Sphingomonadales | Erythrobacteraceae | *Erythrobacter litoralis* |
| 640427126 | Proteobacteria | Alphaproteobacteria | Sphingomonadales | Sphingomonadaceae | *Novosphingobium aromaticivorans* |
| 2558860262 | Proteobacteria | Alphaproteobacteria | Sphingomonadales | Sphingomonadaceae | *Sphingomonas sanxanigenens* |
| 2548877156 | Proteobacteria | Betaproteobacteria | Burkholderiales | Alcaligenaceae | *Advenella mimigardefordensis* |
| 637000053 | Proteobacteria | Betaproteobacteria | Burkholderiales | Burkholderiaceae | *Burkholderia xenovorans* |
| 640427129 | Proteobacteria | Betaproteobacteria | Burkholderiales | Burkholderiaceae | *Polynucleobacter necessarius* |
| 650716004 | Proteobacteria | Betaproteobacteria | Burkholderiales | Comamonadaceae | *Acidovorax avenae* |
| 649633106 | Proteobacteria | Betaproteobacteria | Burkholderiales | Comamonadaceae | *Variovorax paradoxus* |
| 644736388 | Proteobacteria | Betaproteobacteria | Methylophilales | Methylophilaceae | *Methylotenera mobilis* |
| 649633068 | Proteobacteria | Betaproteobacteria | Methylophilales | Methylophilaceae | *Methylovorus sp.* |
| 643692026 | Proteobacteria | Betaproteobacteria | Neisseriales | Neisseriaceae | *Laribacter hongkongensis* |
| 650716066 | Proteobacteria | Betaproteobacteria | Nitrosomonadales | Nitrosomonadaceae | *Nitrosomonas sp.* |
| 637000088 | Proteobacteria | Betaproteobacteria | Rhodocyclales | Rhodocyclaceae | *Dechloromonas aromatica* |
| 643692051 | Proteobacteria | Betaproteobacteria | Rhodocyclales | Rhodocyclaceae | *Thauera aminoaromatica* |
| 2623620903 | Proteobacteria | Deltaproteobacteria | Bdellovibrionales | Bdellovibrionaceae | *Bdellovibrio bacteriovorus* |
| 2623620702 | Proteobacteria | Deltaproteobacteria | Desulfuromonadales | Geobacteraceae | *Geobacter sulfurreducens* |
| 646311933 | Proteobacteria | Deltaproteobacteria | Myxococcales | Kofleriaceae | *Haliangium ochraceum* |
| 637000007 | Proteobacteria | Deltaproteobacteria | Myxococcales | Myxococcaceae | *Anaeromyxobacter dehalogenans* |
| 2623620856 | Proteobacteria | Deltaproteobacteria | Myxococcales | Myxococcaceae | *Myxococcus xanthus* |
| 2623620824 | Proteobacteria | Deltaproteobacteria | Syntrophobacterales | Syntrophaceae | *Syntrophus aciditrophicus* |
| 639633063 | Proteobacteria | Deltaproteobacteria | Syntrophobacterales | Syntrophobacteraceae | *Syntrophobacter fumaroxidans* |
| 2504136012 | Proteobacteria | Gammaproteobacteria | Alteromonadales | Alteromonadaceae | *Alteromonas sp.* |
| 2511231080 | Proteobacteria | Gammaproteobacteria | Alteromonadales | Alteromonadaceae | *Glaciecola nitratireducens* |
| 641522648 | Proteobacteria | Gammaproteobacteria | Alteromonadales | Shewanellaceae | *Shewanella halifaxensis* |
| 646564502 | Proteobacteria | Gammaproteobacteria | Chromatiales | Chromatiaceae | *Allochromatium vinosum* |
| 639633026 | Proteobacteria | Gammaproteobacteria | Chromatiales | Ectothiorhodospiraceae | *Halorhodospira halophila* |
| 643348585 | Proteobacteria | Gammaproteobacteria | Chromatiales | Ectothiorhodospiraceae | *Thioalkalivibrio sulfidophilus* |
| 646862326 | Proteobacteria | Gammaproteobacteria | Enterobacteriales | Enterobacteriaceae | *Escherichia coli* |
| 650716084 | Proteobacteria | Gammaproteobacteria | Enterobacteriales | Enterobacteriaceae | *Salmonella bongori* |
| 643348531 | Proteobacteria | Gammaproteobacteria | Legionellales | Coxiellaceae | *Coxiella burnetii* |
| 2623620935 | Proteobacteria | Gammaproteobacteria | Legionellales | Legionellaceae | *Legionella pneumophila* |
| 2509601034 | Proteobacteria | Gammaproteobacteria | Lysobacterales | Lysobacteraceae | *Frateuria aurantia* |
| 641522602 | Proteobacteria | Gammaproteobacteria | Pseudomonadales | Moraxellaceae | *Acinetobacter baumannii* |
| 2623620882 | Proteobacteria | Gammaproteobacteria | Pseudomonadales | Moraxellaceae | *Acinetobacter baylyi* |
| 637000221 | Proteobacteria | Gammaproteobacteria | Pseudomonadales | Pseudomonadaceae | *Pseudomonas fluorescens* |
| 640427131 | Proteobacteria | Gammaproteobacteria | Pseudomonadales | Pseudomonadaceae | *Pseudomonas mendocina* |
| 2561511166 | Proteobacteria | Gammaproteobacteria | Xanthomonadales | Xanthomonadaceae | *Xanthomonas citri* |
| 641522643 | Verrucomicrobia | Opitutae | unclassified | Opitutaceae | *Opitutus terrae* |
| 642555104 | Verrucomicrobia | Verrucomicrobiae | Verrucomicrobiales | Verrucomicrobiaceae | *Akkermansia muciniphila* |

**Table S4. Selected soil properties.** Mean (± SE) soil characteristics. ANOVAs test whether each characteristic was affected by soil type, treatments, and all interactions. For each characteristic, means with the same letter are not significantly different at α = 0.05. “Harsh” refers to the harsh serpentine soil, “Lush” refers to the lush serpentine soil, and “Non” refers to the non-serpentine soil.

|  | **Soil** | **Control** | **+ Nutrients** | **+ Precipitation** | **+ Nutrients + Precipitation** | **ANOVA results for all *p* < 0.10** |
| --- | --- | --- | --- | --- | --- | --- |
| Organic matter (%) | Harsh | 2.44 ± 0.18 b | 2.34 ± 0.18 b | 2.24 ± 0.19 b | 2.59 ± 0.13 b | Soil: *F*_2,112_= 95.2, *p* < 0.0001  Nutrients: *F*_1,112_= 9.61, *p* = 0.0024 |
|  | Lush | 3.85 ± 0.16 a | 4.36 ± 0.16 a | 3.85 ± 0.22 a | 4.48 ± 0.14 a |  |
|  | Non | 3.80 ± 0.15 a | 4.05 ± 0.31 a | 3.89 ± 0.15 a | 4.13 ± 0.25 a |  |
| Dissolved  organic C  (mg / kg dry soil) | Harsh | 117.5 ± 16.0 bcde | 221.9 ± 33.3 a | 72.1 ± 15.7 defg | 112.1 ± 35.7 cdef | Soil: *F*_2,112_= 6.72, *p* = 0.0018  Precip: *F*_1,112_= 129.3, *p* < 0.0001  Nutrients: *F*_1,112_= 8.01, *p* = 0.0055  Soil x Nutr: *F*_2,112_= 4.09, *p* = 0.019  Precip x Nutr: *F*_1,112_= 5.55, *p* = 0.020 |
|  | Lush | 120.9 ± 15.9 bcd | 141.8 ± 12.5 abc | 45.7 ± 4.5 fg | 40.3 ± 6.7 g |  |
|  | Non | 141.9 ± 10.7 abc | 180.2 ± 17.7 ab | 57.3 ± 4.6 efg | 52.0 ± 3.8 fg |  |
| NH_4_-N  (mg / kg dry soil) | Harsh | 1.80 ± 0.21 bcd | 10.76 ± 2.24 a | 0.53 ± 0.10 d | 1.51 ± 0.24 bcd | Precip: *F*_1,112_=170.9, *p* < 0.0001  Nutrients: *F*_1,112_=115.7 , *p* < 0.0001  Precip x Nutr: *F*_1,112_= 51.9, *p* < 0.0001 |
|  | Lush | 2.22 ± 0.19 bc | 12.77 ± 2.29 a | 1.33 ± 0.87 cd | 1.54 ± 0.30 bcd |  |
|  | Non | 3.19 ± 0.21 b | 9.57 ± 1.07 a | 1.49 ± 0.32 bcd | 2.05 ± 0.33 bcd |  |
| Olsen P  (mg / kg dry soil) | Harsh | 12.29 ± 1.31 c | 29.22 ± 1.99 ab | 12.30 ± 1.10 c | 24.40 ± 1.64 b | Precip: *F*_1,112_= 14.2, *p* = 0.0003  Nutrients: *F*_1,112_= 423.1, *p* < 0.0001  Soil x Nutr: *F*_2,112_= 3.57, *p* = 0.031  Precip x Nutr: *F*_1,112_= 21.4, *p* < 0.0001 |
|  | Lush | 9.38 ± 0.89 c | 36.14 ± 2.50 a | 9.23 ± 0.85 c | 22.28 ± 2.26 b |  |
|  | Non | 10.29 ± 1.10 c | 30.07 ± 3.04 ab | 10.33 ± 0.83 c | 22.11 ± 1.57 b |  |
| K  (mg / kg dry soil) | Harsh | 62.80 ± 10.22 d | 172.01 ± 26.94 bc | 60.73 ± 13.60 d | 145.69 ± 8.65 c | Soil: *F*_2,112_= 30.9, *p* < 0.0001  Precip: *F*_1,112_= 3.90, *p* = 0.051  Nutrients: *F*_1,112_= 91.5, *p* < 0.0001 |
|  | Lush | 177.0 ± 19.52 bc | 272.4 ± 23.53 a | 147.4 ± 13.78 c | 246.9 ± 26.63 ab |  |
|  | Non | 166.7 ± 6.05 bc | 246.0 ± 20.33 ab | 155.0 ± 13.06 c | 217.3 ± 16.4 abc |  |
| pH | Harsh | 7.01 ± 0.08 abc | 6.90 ± 0.07 cd | 7.29 ± 0.07 a | 7.10 ± 0.04 abc | Soil: *F*_2,112_= 76.0, *p* < 0.0001  Precip: *F*_1,112_= 81.5, *p* < 0.0001  Nutrients: *F*_1,112_= 13.1, *p* = 0.0004  Soil x Precip: *F*_2,112_= 7.74, *p* = 0.0007  Soil x Precip x Nutrients:  *F*_2,112_= 2.87, *p* = 0.061 |
|  | Lush | 6.94 ± 0.06 cd | 6.93 ± 0.06 cd | 7.27 ± 0.03 ab | 7.00 ± 0.04 bc |  |
|  | Non | 6.24 ± 0.06 e | 6.11 ± 0.07 e | 6.67 ± 0.07 d | 6.69 ± 0.07 d |  |
| Soil moisture at sampling  (g H_2_O / g dry soil) | Harsh | 0.028 ± 0.004 g | 0.025 ± 0.003 g | 0.073 ± 0.008 de | 0.095 ± 0.005 cd | Soil: *F*_2,112_= 47.5, *p* < 0.0001  Precip: *F*_1,112_= 570.9, *p* < 0.0001  Nutrients: *F*_1,112_= 15.9, *p* = 0.0001  Soil x Precip: *F*_2,112_= 7.11, *p* = 0.0012  Precip x Nutr: *F*_1,112_= 6.12, *p* = 0.015 |
|  | Lush | 0.045 ± 0.004 fg | 0.054 ± 0.005 ef | 0.124 ± 0.005 ab | 0.145 ± 0.006 a |  |
|  | Non | 0.030 ± 0.003 fg | 0.039 ± 0.004 fg | 0.100 ± 0.006 bc | 0.118 ± 0.008 bc |  |

**Table S5. Linear mixed effects models for community-weighted mean estimated traits, with (A, C) and without (B, D) soil properties as covariates.** Soil properties shown for each trait are those that, when added to the model, caused the main effect of precipitation addition and/or nutrient addition to become non-significant (*p* > 0.05). Such soil properties are candidates for mediating the effect of the treatments on microbial traits, although further experiments to isolate changes in these soil properties would be necessary to firmly establish their role. Ammonium (NH_4_^+^-N) and dissolved organic carbon (DOC) were log-transformed in these analyses to meet linear model assumptions**.**

| **A. Estimated rRNA gene copy number:**  **Treatments only** | | | |
| --- | --- | --- | --- |
| **Factor** | **df** | ***F*** | ***p*** |
| Soil type | 2, 109 | 17.26 | < 0.0001 |
| Precipitation | 1, 109 | 13.54 | 0.0004 |
| Nutrients | 1, 109 | 18.29 | < 0.0001 |
| Soil type x Precipitation | 2, 109 | 1.46 | 0.236 |
| Soil type x Nutrients | 2, 109 | 0.15 | 0.864 |
| Precipitation x Nutrients | 1, 109 | 0.003 | 0.958 |
| Soil type x Precipitation x Nutrients | 2, 109 | 0.13 | 0.882 |

| **B. Estimated rRNA gene copy number:**  **With covariates** | | **P** | | **K** | | **NH_4_^+^-N** | | **DOC** | |
| --- | --- | --- | --- | --- | --- | --- | --- | --- | --- |
| **Factor** | **df** | ***F*** | ***p*** | ***F*** | ***p*** | ***F*** | ***p*** | ***F*** | ***p*** |
| Covariate | 1, 108 | 24.93 | < 0.0001 | 59.73 | < 0.0001 | 41.95 | < 0.0001 | 5.33 | 0.023 |
| Soil type | 2, 108 | 17.47 | < 0.0001 | 3.60 | 0.031 | 16.70 | < 0.0001 | 20.62 | < 0.0001 |
| Precipitation | 1, 108 | 7.72 | 0.007 | 9.27 | 0.003 | 0.73 | 0.394 | 2.59 | 0.110 |
| Nutrients | 1, 108 | 1.34 | 0.249 | 2.60 | 0.110 | 1.29 | 0.260 | 16.91 | 0.000 |
| Soil x Precip | 2, 108 | 1.18 | 0.312 | 1.32 | 0.272 | 0.96 | 0.386 | 1.40 | 0.252 |
| Soil x Nutrients | 2, 108 | 0.05 | 0.952 | 0.18 | 0.838 | 0.86 | 0.427 | 0.21 | 0.807 |
| Precip x Nutrients | 1, 108 | 0.26 | 0.610 | 0.03 | 0.868 | 0.64 | 0.427 | 0.00 | 0.960 |
| Soil x Precip x Nutrients | 2, 108 | 0.05 | 0.955 | 0.25 | 0.783 | 0.02 | 0.977 | 0.12 | 0.891 |

| **C. Estimated genome size:**  **Treatments only** |  |  | |
| --- | --- | --- | --- |
| **Factor** | **df** | ***F*** | ***p*** |
| Soil type | 2, 109 | 70.40 | <.0001 |
| Precipitation | 1, 109 | 10.85 | 0.001 |
| Nutrients | 1, 109 | 3.32 | 0.071 |
| Soil type x Precipitation | 2, 109 | 2.53 | 0.084 |
| Soil type x Nutrients | 2, 109 | 0.59 | 0.555 |
| Precipitation x Nutrients | 1, 109 | 0.09 | 0.759 |
| Soil type x Precipitation x Nutrients | 2, 109 | 0.15 | 0.860 |

| **D. Estimated genome size:**  **With covariates** | | **P** | | **K** | | **NH_4_^+^-N** | | **DOC** | | **pH** | | **Organic matter** | |
| --- | --- | --- | --- | --- | --- | --- | --- | --- | --- | --- | --- | --- | --- |
| **Factor** | **df** | ***F*** | ***p*** | ***F*** | ***p*** | ***F*** | ***p*** | ***F*** | ***p*** | ***F*** | ***p*** | ***F*** | ***p*** |
| Covariate | 1, 108 | 3.52 | 0.063 | 65.71 | <0.0001 | 36.35 | <0.0001 | 9.35 | 0.003 | 82.94 | <0.0001 | 99.42 | <0.0001 |
| Soil type | 2, 108 | 70.49 | <0.0001 | 35.54 | <0.0001 | 56.75 | <0.0001 | 73.83 | <0.0001 | 38.04 | <0.0001 | 19.94 | <0.0001 |
| Precipitation | 1, 108 | 8.46 | 0.004 | 8.53 | 0.004 | 0.01 | 0.908 | 0.02 | 0.883 | 2.00 | 0.160 | 13.42 | 0.0004 |
| Nutrients | 1, 108 | 0.70 | 0.403 | 0.10 | 0.750 | 0.66 | 0.417 | 1.87 | 0.174 | 1.50 | 0.223 | 0.65 | 0.421 |
| Soil x Precip | 2, 108 | 2.48 | 0.088 | 2.37 | 0.099 | 1.67 | 0.193 | 1.93 | 0.149 | 3.73 | 0.027 | 2.90 | 0.059 |
| Soil x Nutr | 2, 108 | 0.62 | 0.540 | 0.76 | 0.471 | 1.35 | 0.264 | 1.67 | 0.193 | 0.73 | 0.485 | 0.19 | 0.828 |
| Precip x Nutr | 1, 108 | 0.05 | 0.828 | 0.31 | 0.582 | 2.62 | 0.109 | 0.82 | 0.367 | 0.01 | 0.920 | 0.01 | 0.931 |
| Soil x Precip x Nutr | 2, 108 | 0.14 | 0.871 | 0.07 | 0.934 | 0.24 | 0.787 | 0.24 | 0.789 | 0.16 | 0.851 | 0.30 | 0.738 |

**Table S6.** Trait estimate means and means of estimate standard errors by phylum. Phyla are sorted in decreasing order of total relative abundance in the experimental data set. Phyla with ≤ 0.01% relative abundance are not shown.

| **Phylum**  **(broken out by class for Proteobacteria)** | **Mean of rRNA gene copy number estimates** | **Mean of rRNA gene copy number estimate SEs** | **Mean of genome size estimates** | **Mean of genome size estimate SEs** |
| --- | --- | --- | --- | --- |
| Actinobacteria | 2.10 | 0.502 | 4.66 | 0.362 |
| Acidobacteria | 1.64 | 0.534 | 3.81 | 0.532 |
| Verrucomicrobia | 1.86 | 0.771 | 6.46 | 0.458 |
| Alphaproteobacteria | 2.09 | 0.479 | 3.80 | 0.435 |
| Bacteroidetes | 3.22 | 0.527 | 5.33 | 0.503 |
| Betaproteobacteria | 2.80 | 0.339 | 4.07 | 0.447 |
| Gemmatimonadetes | 1.70 | 0.523 | 3.82 | 0.506 |
| Planctomycetes | 2.85 | 0.712 | 5.19 | 0.706 |
| Chloroflexi | 2.29 | 0.700 | 3.10 | 0.550 |
| Deltaproteobacteria | 2.29 | 0.472 | 5.96 | 0.501 |
| Crenarchaeota | 1.00 | 0.578 | 2.34 | 0.463 |
| Gammaproteobacteria | 2.56 | 0.513 | 3.24 | 0.351 |
| Armatimonadetes | 2.77 | 0.769 | 3.17 | 0.540 |
| Elusimicrobia | 1.11 | 0.644 | 1.61 | 0.560 |
| Chlorobi | 1.24 | 0.552 | 3.33 | 0.503 |
| Nitrospirae | 1.55 | 0.651 | 3.48 | 0.546 |
| Cyanobacteria | 2.43 | 0.504 | 3.30 | 0.547 |
| FBP | 6.41 | 0.520 | 3.27 | 0.396 |
| Tenericutes | 2.64 | 0.485 | 2.03 | 0.481 |
| TM7 | 1.20 | 0.930 | 1.15 | 0.570 |
| TM6 | 1.07 | 1.033 | 1.15 | 0.604 |
| Euryarchaeota | 1.06 | 0.618 | 1.04 | 0.775 |
| Chlamydiae | 2.20 | 0.411 | 2.55 | 0.325 |
| BRC1 | 2.35 | 0.481 | 2.73 | 0.536 |
| Fibrobacteres | 2.25 | 0.711 | 3.31 | 0.904 |
| OD1 | 1.68 | 0.762 | 0.81 | 0.692 |
| WS3 | 2.20 | 0.540 | 2.37 | 0.547 |
| OP3 | 1.80 | 0.693 | 1.26 | 0.651 |
| Firmicutes | 7.14 | 0.468 | 4.23 | 0.380 |
| [Parvarchaeota] | 1.25 | 0.785 | 0.69 | 1.028 |

# Supplementary Figures

**Figure S1.** **OTUs that increased in relative abundance in response to both treatments differed in estimated rRNA gene copy number, on average, from OTUs that decreased in relative abundance**. Bars represent unweighted mean estimated rRNA gene copy number values across all “increaser” and “decreaser” OTUs for each treatment (see Methods for how “increasers” and “decreasers” were defined). Error bars show 1 SE below mean and 1 SE above mean. In response to nutrient addition, estimated rRNA gene copy number was greater among OTUs that increased than among OTUs that decreased, on average. In response to precipitation addition, the opposite pattern was observed: estimated rRNA gene copy number was smaller among OTUs that increased than among OTUs that decreased, on average.


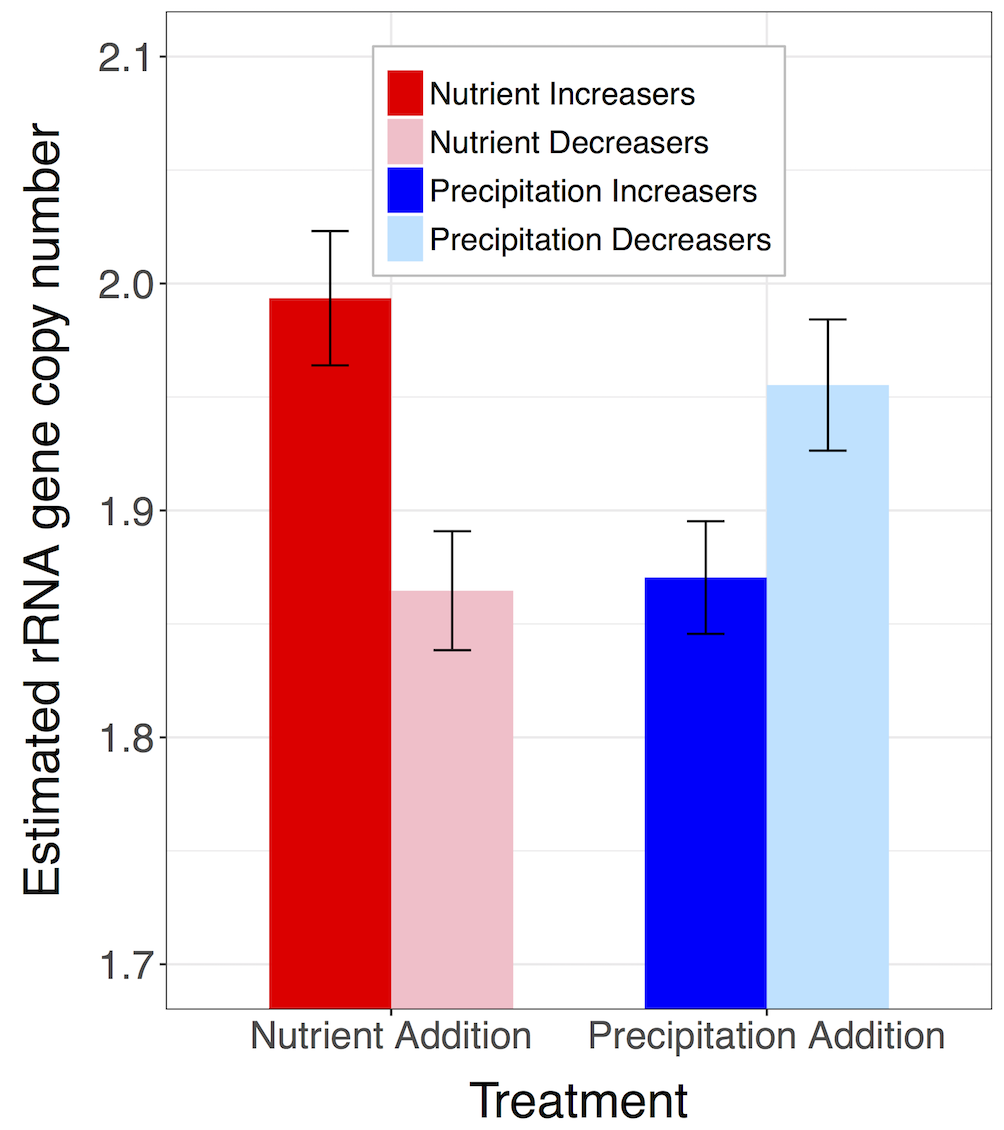


**Figure S2. (A) OTUs that increased in relative abundance in response to both treatments differed in estimated genome size, on average, from OTUs that decreased in relative abundance**. Bars represent unweighted mean estimated genome size values across all “increaser” and “decreaser” OTUs for each treatment (see Methods for how “increasers” and “decreasers” were defined). Error bars show 1 SE below mean and 1 SE above mean. In response to nutrient addition, estimated genome size was greater among OTUs that increased than among OTUs that decreased, on average. In response to precipitation addition, the opposite pattern was observed: estimated genome size was smaller among OTUs that increased than among OTUs that decreased, on average. **(B)** **OTUs that increased in relative abundance in response to precipitation addition differed in estimated genome size, on average, from OTUs that decreased in relative abundance on two of the three soil types.** We explored precipitation increasers and decreasers by soil type due to the borderline significant interaction between these two factors in our linear mixed effects model for community-weighted mean estimated genome size. On the lush serpentine and non-serpentine soil, estimated genome size was smaller among OTUs that increased than among OTUs that decreased (*t*_1467_ = 4.19, *p* < 0.0001 and *t*_1594_ = 2.65, *p* = 0.008, respectively). On the harsh serpentine soil, a similar trend was observed (*t*_1625_ = 1.74, *p* = 0.081).


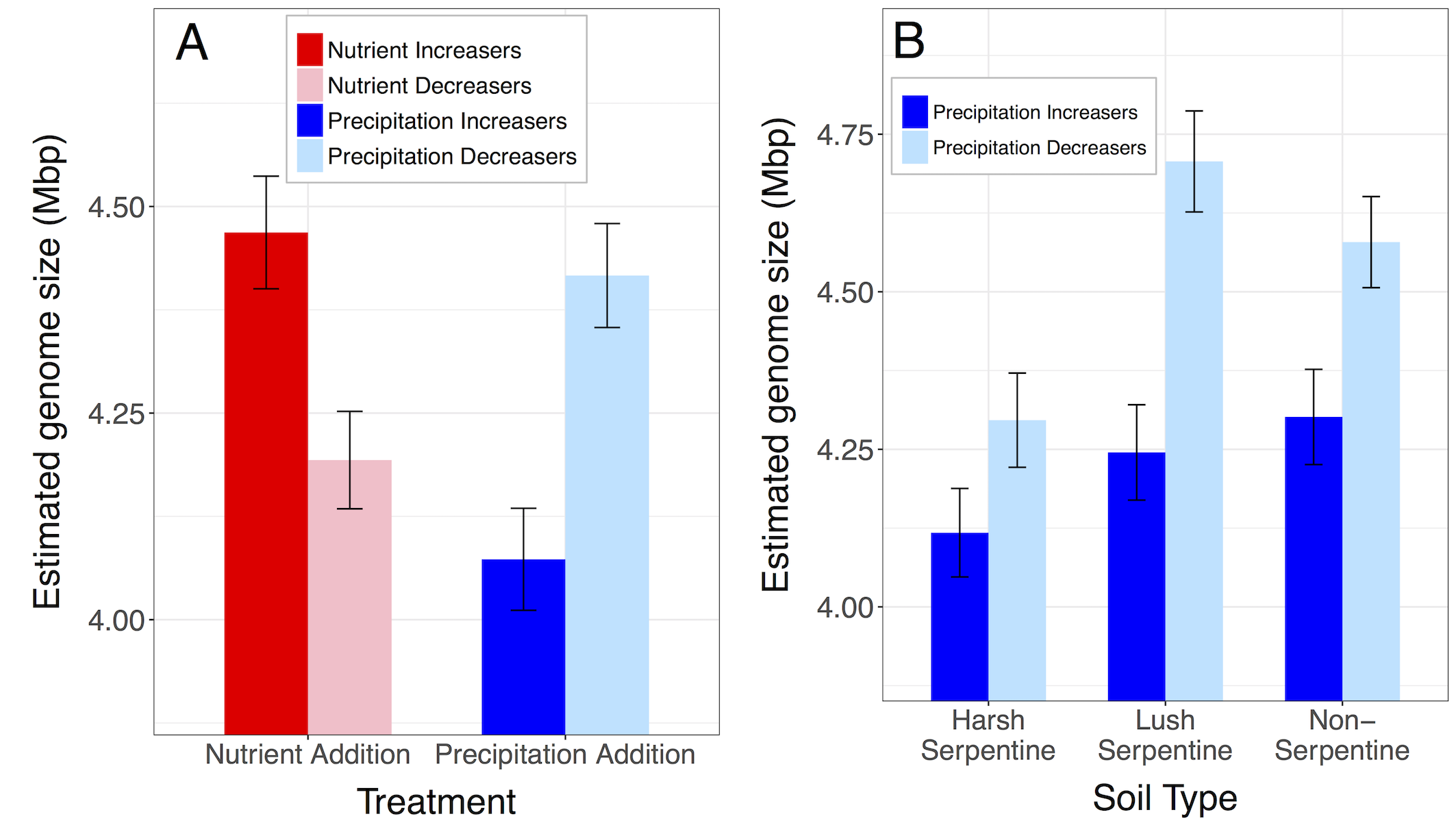


**Figure S3.** **Community-weighted mean estimated rNA gene copy number for all combinations of soil type and treatments**. Error bars show 1 SE below mean and 1 SE above mean. Bars sharing a letter are not significantly different (at α = 0.05). The *y*-axis is scaled to highlight variation among soils and treatments, as it is not possible to have 0 rRNA gene copies. Although few treatments differed from one another within soils, the main effects of both nutrient addition and precipitation addition were significant in the full linear mixed effects model. The similar effects of the treatments across soils contributed to these significant main effects, despite relatively small effect sizes.


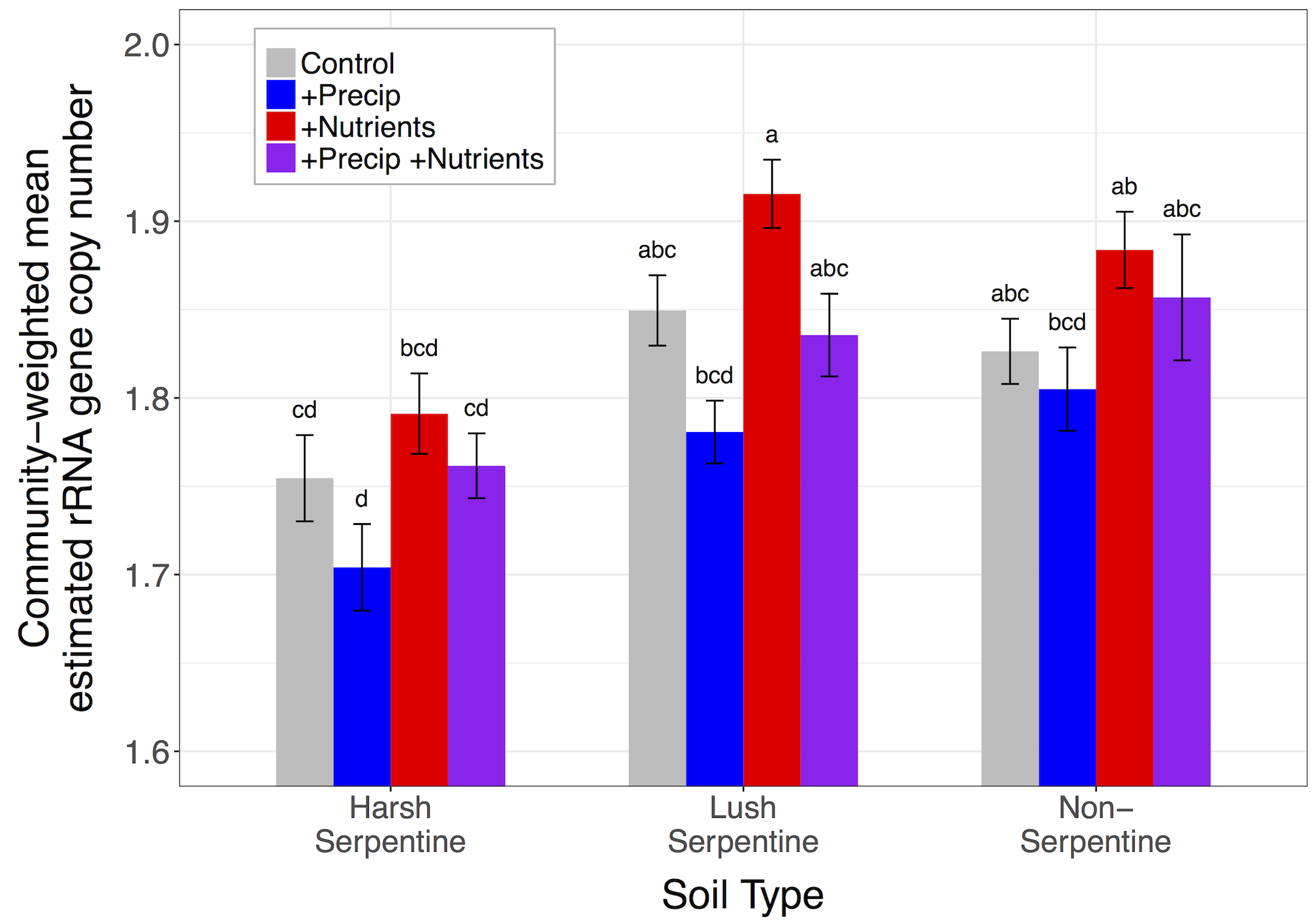


**Figure S4.** **Community-weighted mean estimated genome size for all combinations of soil type and treatments**. Error bars show 1 SE below mean and 1 SE above mean. Bars sharing a letter are not significantly different (at α = 0.05). The *y*-axis is scaled to highlight variation among soils and treatments, as it is not possible to have a 0 Mbp genome. As for estimated rRNA gene copy number, although few treatments differed from one another within soils, the main effect of precipitation addition was significant and the main effect of nutrient addition was marginally significant (*p* = 0.071) in the full linear mixed effects model. For estimated genome size, the effects of precipitation addition diverged slightly across soils, having greatest effect in the lush serpentine soil. Still, soils also showed some similarities in their responses, contributing to significant main effects despite relatively small effect sizes.


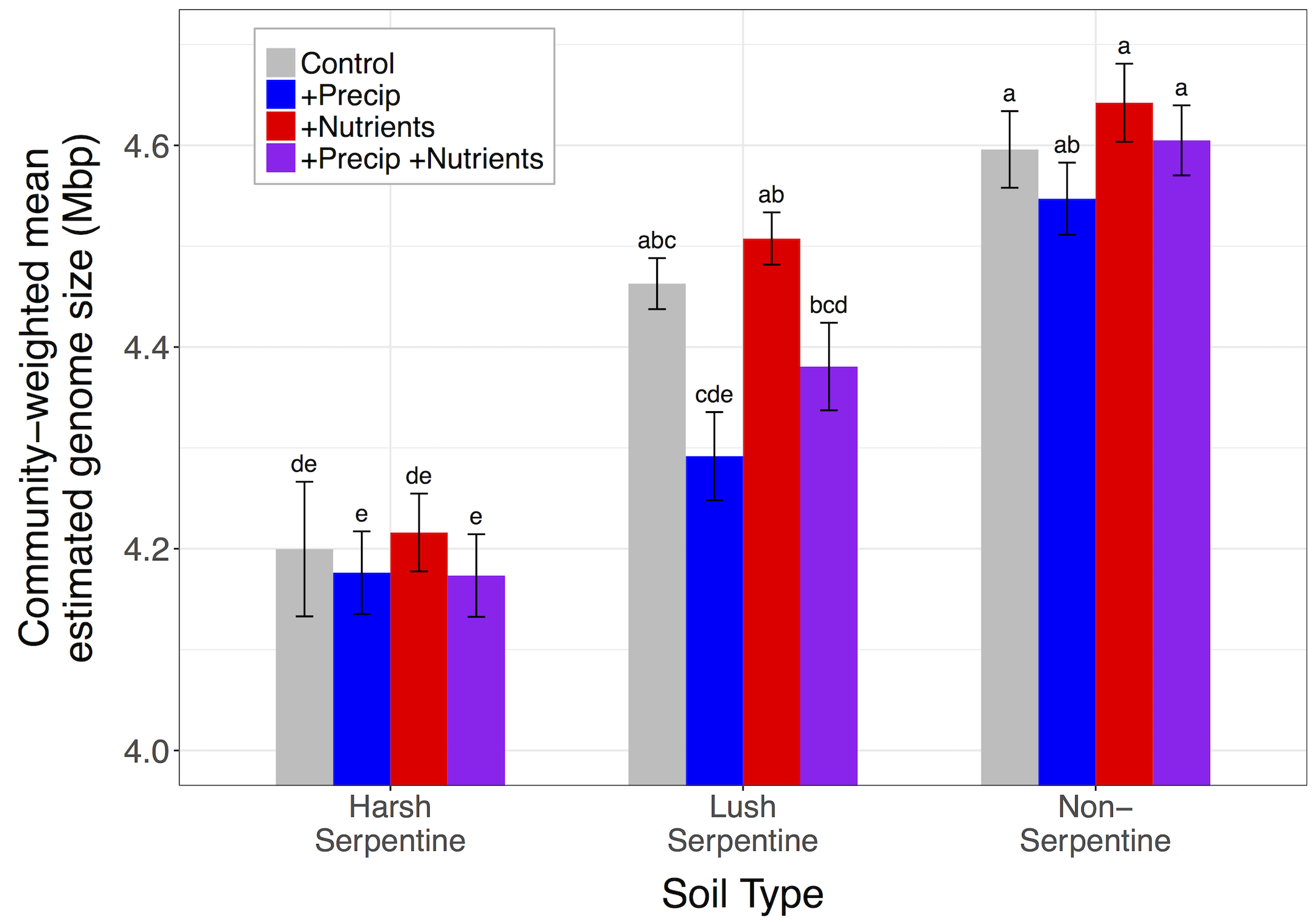


# References

Blomberg, S. P., Garland, T., Ives, A. R., Garland Jr, T., and Ives, A. R. (2003). Testing for phylogenetic signal in comparative data: behavioral traits are more labile. *Evolution (N. Y).* 57, 717–745. doi:10.1111/j.0014-3820.2003.tb00285.x.

Caporaso, J. G., Bittinger, K., Bushman, F. D., DeSantis, T. Z., Andersen, G. L., and Knight, R. (2010a). PyNAST: A flexible tool for aligning sequences to a template alignment. *Bioinformatics* 26, 266–267. doi:10.1093/bioinformatics/btp636.

Caporaso, J. G., Kuczynski, J., Stombaugh, J., Bittinger, K., Bushman, F. D., Costello, E. K., et al. (2010b). QIIME allows analysis of high-throughput community sequencing data. *Nat. Methods* 7, 335–336. doi:10.1038/nmeth0510-335.

Fierer, N., Barberán, A., and Laughlin, D. C. (2014). Seeing the forest for the genes: using metagenomics to infer the aggregated traits of microbial communities. *Front. Microbiol.* 5, 1–6. doi:10.3389/fmicb.2014.00614.

Fritz, S. A., and Purvis, A. (2010). Selectivity in mammalian extinction risk and threat types: A new measure of phylogenetic signal strength in binary traits. *Conserv. Biol.* 24, 1042–1051. doi:10.1111/j.1523-1739.2010.01455.x.

Goberna, M., and Verdú, M. (2016). Predicting microbial traits with phylogenies. *ISME J.* 10, 959–967. doi:10.1038/ismej.2015.171.

Huntemann, M., Ivanova, N. N., Mavromatis, K., Tripp, H. J., Paez-Espino, D., Palaniappan, K., et al. (2015). The Standard Operating Procedure of the DOE-JGI Microbial Genome Annotation Pipeline ( MGAP v . 4 ) Keywords. *Stand. Genomic Sci.*, 1–6. doi:10.1186/s40793-015-0077-y.

Kembel, S. W., Wu, M., Eisen, J. A., and Green, J. L. (2012). Incorporating 16S gene copy number information improves estimates of microbial diversity and abundance. *PLoS Comput. Biol.* 8, e1002743. doi:10.1371/journal.pcbi.1002743.

Krause, S., Le Roux, X., Niklaus, P. a, Van Bodegom, P. M., Lennon, J. T., Bertilsson, S., et al. (2014). Trait-based approaches for understanding microbial biodiversity and ecosystem functioning. *Front. Microbiol.* 5, 251. doi:10.3389/fmicb.2014.00251.

Lauro, F. M., McDougald, D., Thomas, T., Williams, T. J., Egan, S., Rice, S., et al. (2009). The genomic basis of trophic strategy in marine bacteria. *Proc. Natl. Acad. Sci. U. S. A.* 106, 15527–33. doi:10.1073/pnas.0903507106.

Madigan, M. T., Martinko, J. M., Stahl, D., and Clark, D. P. (2010). *Brock Biology of Microorganisms*. 13th ed. San Francisco, CA: Benjamin Cummings.

Markowitz, V. M., Chen, I. M. A., Palaniappan, K., Chu, K., Szeto, E., Grechkin, Y., et al. (2012). IMG: The integrated microbial genomes database and comparative analysis system. *Nucleic Acids Res.* 40, 115–122. doi:10.1093/nar/gkr1044.

Mavromatis, K., Land, M. L., Brettin, T. S., Quest, D. J., Copeland, A., Clum, A., et al. (2012). The Fast Changing Landscape of Sequencing Technologies and Their Impact on Microbial Genome Assemblies and Annotation. *PLoS One* 7, e48837. doi:10.1371/journal.pone.0048837.

Orme, D., Freckleton, R., Thomas, G., Petzoldt, T., Fritz, S., Isaac, N., et al. (2013). caper: Comparative Analyses of Phylogenetics and Evolution in R. R package version 0.5.2. https://CRAN.R-project.org/package=caper.

Pagel, M. (1999). Inferring the historical patterns of biological evolution. *Nature* 401, 877–884. doi:10.1038/44766.

Revell, L. J. (2012). phytools: An R package for phylogenetic comparative biology (and other things). *Methods Ecol. Evol.* 3, 217–223. doi:10.1111/j.2041-210X.2011.00169.x.

Stamatakis, A. (2014). RAxML version 8: A tool for phylogenetic analysis and post-analysis of large phylogenies. *Bioinformatics* 30, 1312–1313. doi:10.1093/bioinformatics/btu033.
